# Cobaltocenylidene: A Mesoionic Metalloceno Carbene, Stabilized in a Gold(III) Complex

**DOI:** 10.1002/chem.201800147

**Published:** 2018-02-05

**Authors:** Stefan Vanicek, Maren Podewitz, Christopher Hassenrück, Michael Pittracher, Holger Kopacka, Klaus Wurst, Thomas Müller, Klaus R. Liedl, Rainer F. Winter, Benno Bildstein

**Affiliations:** ^1^ Institute of General, Inorganic and Theoretical Chemistry University of Innsbruck Center for Chemistry and Biomedicine Innrain 80–82 6020 Innsbruck Austria; ^2^ Department of Chemistry University of Konstanz Universitätsstrasse 10 784557 Konstanz Germany; ^3^ Institute of Organic Chemistry University of Innsbruck Center for Chemistry and Biomedicine Innrain 80–82 6020 Innsbruck Austria

**Keywords:** cobalt, density functional calculations, gold, mesoionic carbenes, sandwich complexes

## Abstract

Oxidative addition of cobaltoceniumdiazonium bis(hexafluoridophosphate) with (pseudo)halide aurates gave gold(III) complexes containing zwitterionic cobaltoceniumide as a ligand. Its selenium derivative, cobaltoceniumselenolate, was obtained by an electrophilic aromatic substitution reaction of iodocobaltocenium iodide with Na_2_Se. Spectroscopic and structural data in combination with DFT calculations showed that this cobaltocenylidene species is a mesoionic carbene quite different from common N‐heterocyclic carbenes. Its ligand properties (TEP, singlet‐triplet gap, nucleophilicity, π‐acidity, Brønsted basicity) are in part comparable to those of cyclic (amino)(alkyl/aryl)carbenes. Electrochemistry data showed that the mesoionic cobaltoceniumides are more electron‐rich than their parent ferrocenes. The reversible reduction of the tricyanido gold complex appears 50 mV negative of the cobaltocenium/cobaltocene couple, whereas that of the selenide derivative is shifted cathodically by 550 mV.

Since the seminal publication on the first stable and crystalline imidazolylidene by Arduengo and co‐workers in 1991,^1^ N‐heterocyclic carbenes (NHCs) in their many versions^2^ continue to attract ever increasing interest in chemical research. Herein, we would like to report a gold complex of a conceptually new type of a mesoionic carbene (MIC)^2^ based on the chemically very stable cobaltocenium moiety. Scheme 1 shows the principal resonance structures of NHCs in comparison to those of a “cobaltocenylidene” CcC (“Cc” stands for cobaltocenyl and “C” for carbene), that may be described either as a cobaltoceniumide zwitterion containing a Co^III^ metal center (structure **A**), or as an η^5^‐cyclopentadienyl‐η^4^‐cyclopentadienylidene‐Co^I^ complex (structure **B**). Both structures are diamagnetic 18 valence electron singlet carbene species. However, the best description of the electronic structure of CcC will turn out (see below), it is an interesting nucleophilic carbene with clearly distinctly different stereoelectronic properties in comparison to standard NHCs: axial steric shielding instead of peripheral substituents in combination with a redox‐active metal center, potentially capable of undergoing reversible Co^III^/Co^II^ and Co^II^/Co^I^ redox processes.

**Scheme 1:**
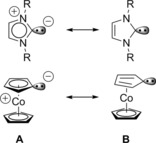
Comparison of resonance structures of NHCs and cobaltocenylidene (CcC).

The reaction of a mixture of cobaltoceniumdiazonium bis(hexafluoridophosphate) (**1**),^3^ potassium dicyanidoaurate, and potassium cyanide in nitromethane solution resulted in an oxidative addition at gold(I) with concomitant N_2_ evolution, affording the cobaltocenylidene complex (CcC)Au(CN)_3_ (**2**) as an air‐stable, yellow solid (Scheme 2 and the Supporting Information). This approach was inspired by recent publications on similar (photocatalyzed) reactions of simple organic aryldiazonium salts with other common gold(I) synthons.^4^ However, in our case, we had to use K[Au(CN)_2_] as gold(I) substrate containing small cyanido ligands that allow unhindered access of the oxidizing reactant, thereby greatly facilitating the desired oxidative addition. That reaction is also supported by the polar solvent nitromethane, which turns out to be the only medium compatible with the highly reactive dicationic cobaltoceniumdiazonium salt.^3^ In addition, one equivalent of KCN was required to provide a fourth ligand for a neutral Au^III^ species. The choice of the strong‐field ligand CN^−^ was also dictated by the high stability of [Au(CN)_2_]^−^, Au(CN)_3_, and [Au(CN)_4_]^−^.^5^ With a weaker ligand, such as Cl^−^, the corresponding complex (CcC)AuCl_3_ (**3**) was obtained in a synthetically unfeasible manner due to severe problems in its purification and separation from by‐products (see the Supporting Information). The highly reactive cobaltoceniumdiazonium bis(hexafluoridophosphate) (**1**) containing N_2_ as the best leaving group turned out as the optimal CcC synthon. Attempted oxidative additions of the less reactive iodocobaltocenium hexafluoridophosphate^3^ with various d8 metal precursors of Rh^+^ and Pt^2+^ gave mostly no reaction at all or intractable product mixtures. We also note that our earlier attempts of CcC complex formation by thermal extrusion of CO_2_ from late transition‐metal cobaltoceniumcarboxylato complexes by the Pesci reaction^6^ met with failure.^7^ However, we have to give credit to Wadepohl for older work from 1987 on dicobalt “cyclopentadienylidene” complexes [(CcC)Co(Cp)L] (Cp=cyclopentadienyl, L=2 electron donor), which have been obtained by serendipity by a double C−H activation reaction.^8^

**Scheme 2:**
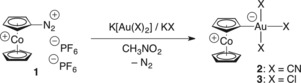
Synthesis of Au^III^ complexes.

Cobaltocenylidene complex **2** is highly polar, very stable both in solution (no hydrolysis in boiling water) and in the solid state (decomposition point: >300 °C). ^1^H and ^13^C NMR spectroscopic signals are in agreement with an undistorted, monofunctionalized cobaltoceniumyl moiety [*δ*(^1^H)=5.67 (pseudo‐t, 2 H), 5.81 (s, 5 H), 5.86 (pseudo‐t, 2 H); *δ*(^13^C)=85.3 (Cp_subst._), 85.4 (Cp_unsubst._), 91.7 ppm (Cp_subst._)] coordinated to a metal center bearing two *cis*‐cyanido ligands [*δ*(^13^C)=120.4 ppm] and one *trans*‐cyanido ligand [*δ*(^13^C)=123.7 ppm] with chemical shifts comparable to those of other simple Au^III^ cyanido complexes.^9^ The carbene carbon of the CcC ligand gives rise to a signal at 113.6 ppm at significantly higher field in comparison to those of other NHC Au^III^ complexes,^[10 ]^indicating much stronger nucleophilicity of the CcC ligand. IR spectroscopy and high‐resolution mass spectrometry data support further the identity of **2** (see the Supporting Information). Unambiguous structural proof for **2** is provided by a high‐quality single‐crystal structure analysis (*R*=1.86 %, Figure 1).

**Figure 1:**
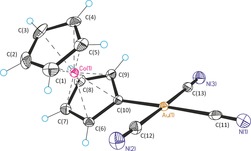
Molecular structure of **2** with thermal ellipsoids at a 50 % probability level. Selected bond lengths [Å] and angles [°]: Au(1)−C(10) 2.038(3), Au(1)−C(11) 2.080(4), Au(1)−C(12) 2.003(4), Au(1)−C(13) 2.013(4), Co(1)−C(10) 2.048(3), Co(1)−C(6) 2.028(3), Co(1)−C(7) 2.029(4), Co(1)−C(8) 2.026(3), Co(1)−C(9) 2.029(3), C(10)−C(9) 1.425(5), C(10)−C(6) 1.421(5), C(9)−C(8) 1.416(5), C(6)−C(7) 1.415(5), C(7)−C(8) 1.419(6); C(11)‐Au(1)‐C(13) 90.66(16), C(11)‐Au(1)‐C(12) 90.99(15), plane [C(6)‐C(10)]‐plane [C(11), C(12), C(13), Au(1)] 34.0(1).

Overall, the molecular structure of **2** shows the expected square‐planar coordination at the Au^III^ center with a tilted cobaltoceniumyl substituent [angle of plane_Cp_ versus plane Au_CN_=34.0(1)°], due to steric repulsion by the *cis*‐cyanido ligands. The carbene carbon–gold bond [C(10)−Au(1)=2.038(3) Å] is quite short, but slightly elongated in comparison to that of other NHC Au^III^ complexes.^10^ The C−C bond lengths of the substituted Cp ring are more or less equal within standard deviations and all five carbon atoms are coplanar, indicating a regular cyclopentadienyl moiety. Only the distance of the carbene carbon to the cobalt center is slightly elongated in comparison to the other C−Co bonds. A rather large difference between the *cis*‐Au−CN bond lengths [Au(1)−C(12)=2.003(4), Au(1)−C(13)=2.013(4)] and the *trans*‐Au−CN distance [Au(1)−C(11)=2.080(4)] indicates a strong thermodynamic *trans* effect of the CcC ligand. The corresponding X‐ray structure of complex **3** (see the Supporting Information) containing chlorido ligands instead of cyanido ligands is very similar. Notably, this bond is even longer (Au−Cl_*trans*_ 2.354 Å in comparison to other [NHC]AuCl_3_ complexes (Au−Cl_*trans*_=2.298–2.325 Å)^10c^ and closely resembles the value in [(H_5_C_6_)_3_P]AuCl_3_ (Au−Cl_*trans*_=2.347 Å)^[11^, see the Supporting Information). Taken all structural data together, the CcC ligand is present as a strongly nucleophilic mesoionic carbene in line with structure **A** in Scheme 1.

To gain further insight into the electronic structure and ligand properties of **2** and of the CcC ligand alone, DFT calculations with two different exchange‐correlation functionals with and without empirical dispersion corrections were performed.^12^ Structural parameters of **2** were in good agreement with experimental data from the single‐crystal diffraction (see the Supporting Information, Table S2). Calculations of molecular electrostatic potential (MEP)^13^ of free carbene CcC (Figure 2) showed a negative (red) MEP at the carbene implying a mesoionic cobaltoceniumide containing an undistorted cobaltocenium group, which is in line with structure **A**. All attempts to converge the electronic structure of **B** converged back to structure **A** (see Scheme 1). The calculated p*K*_a_ of 38.5(±2) in nitromethane (treated as implicit solvent)^14^ is much higher in comparison to that of standard NHCs (p*K*_a_≈24),^2^ indicating CcC to be a much more electron‐donating ligand (see the Supporting Information for details on the calculation). The calculated singlet‐triplet gap is in between 20.0 and 27.0 kcal mol^−1^, depending on the density functional employed.^15^ Thus, it is very small compared to those of NHCs,^2^ cyclic (alkyl)aminocarbenes (CAACs),^16^ and even cyclic (amino)(aryl)carbenes (CAArCs).^17^ However, the triplet species of CcC is the organometallic biradical cobaltocenyl [Cc=η^5^‐(C_5_H_5_)‐η^5^‐C_5_H_4_)Co] containing a cyclopentadienyl radical and a paramagnetic cobalt(II) center, clearly not directly comparable to triplet species of NHCs. To provide a metric for the characterization of CcC in comparison to other NHCs, the Tolman electronic parameter (TEP)^18^ was calculated and a value of 2037.1 cm^−1^ was obtained, thereby ranking CcC with respect to its ligand properties in between CAACs and CAArCs (Scheme 3).

**Figure 2:**
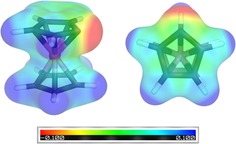
Structure and molecular electrostatic potential (MEP) of CcC. Left: side view; right: top view.

**Scheme 3:**
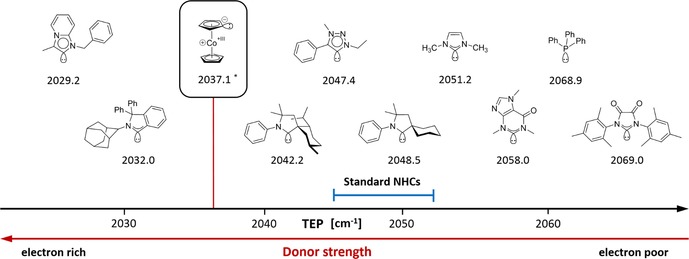
Tolman electronic parameter (TEP)^[a]^ of CcC in comparison to those of other NHCs. [a] TEP data of NHCs were taken from Ref. 18b and Ref. 17; TEP of CcC calculated (see text and the Supporting Information).

Experimental evaluations of the σ‐donor character and the π‐backbonding properties of NHCs are commonly made by measurements of the IR stretching frequencies of their metal carbonyl complexes, [Ni(CO)_3_(NHC)], [RhCl(CO)_2_(NHC)], or [IrCl(CO)_2_(NHC)],^18b^ and by ^31^P or ^77^Se NMR measurements of their phosphinidene derivatives, NHC–P(C_6_H_5_),^16^, ^17^ or selenium adducts, NHC–Se.^19^ Unfortunately, Ni^0^/Rh^I^/Ir^I^ carbonyl complexes and phosphinidene or selenium derivatives containing the CcC ligand are not accessible by an oxidative addition methodology. However, given the fact that cationic cobaltoceniumyl is a strong electron‐accepting aromatic moiety, it was proved to be possible to synthesize CcC–Se adduct (**5**) by an electrophilic aromatic substitution reaction of sodium selenide^20^ with iodocobaltocenium iodide (**4**; Scheme 4).^3^ We note that similar electrophilic aromatic substitution reactions of N/O/S‐nucleophiles with nitro‐pentamethylcobaltocenium hexafluoridophosphate were recently published.^21^ In the course of measuring relevant ^77^Se NMR data of **5** (see below), it turned out that common selenium with its 7.6 % natural abundance of ^77^Se was too insensitive for some of our purposes, necessitating the synthesis of its isotopically enriched analogue (**5 b**) with 99.5 % ^77^Se. Because elemental ^77^Se is only available in the form of unreactive metallic cuttings, quite harsh reaction conditions were required for the synthesis of Na_2_^77^Se (3 days of ultrasonic activation, 1 d heating at reflux on a 10 mg scale) in comparison to the less cumbersome synthesis of Na_2_Se starting from common grey selenium powder (see the Supporting Information).^20^

**Scheme 4:**
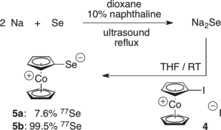
Synthesis of CcC–Se (**5**).

CcC‐Se adducts (**5 a/b**) were obtained from Na_2_Se or Na_2_^77^Se in almost quantitative yields as highly air‐sensitive, dark purple compounds. Single crystals of **5 a** were grown by crystallization from dry acetone, and its molecular structure is depicted in Figure 3. Overall, zwitterionic **5 a** is a regular cobaltocenium compound without relevant distortions. Only the distance of the Se carbon to the cobalt center C(10)−Co(1) is slightly elongated in comparison to the other C−Co bonds. The carbon–selenium bond length C(10)−Se(1) of 1.8613(19) Å is slightly longer in comparison to those of Se adducts of standard NHCs (1.82–1.84 Å),^[19 ]^considerably elongated in comparison to those of the most electron‐poor, strongly π‐acidic diamido NHCs,^22^ and shortened in comparison to a standard carbon–selenium single bond (1.98 Å), indicating CcC to be a more nucleophilic mesoionic carbene in comparison to standard NHCs with slightly reduced π‐acidity.

**Figure 3:**
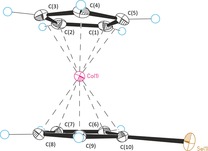
Molecular structure of **5 a** with thermal ellipsoids at 50 % probability level. Selected bond lengths [Å]: Se(1)−C(10) 1.8613(19), Co(1)−C(10) 2.1183(18), Co(1)−C(9) 2.0439(18), Co(1)−C(8) 2.0189(19), Co(1)−C(7) 2.0161(18), Co(1)−C(6) 2.0305(18), C(10)−C(9) 1.443(3), C(9)−C(8) 1.419(3), C(8)−C(7) 1.418(3), C(7)−C(6) 1.426(3), C(6)−C(10) 1.436(3).

Spectroscopically, cobaltoceniumselenolates (**5 a/b**) were characterized by ^1^H/^13^C/^77^Se NMR, IR, and mass spectrometry (see the Supporting Information). The strong selenium–carbon dipole is clearly evident by its intense IR stretching vibration observed at 828 cm^−1^. Most important, NMR results comprise the following data: The chemical shift of the selenolate carbon, *δ*(^13^C)=129.4 ppm, is shifted by 47.3 ppm to lower field in comparison to the resonance in unsubstituted cobaltocenium CcH^+^, corroborating the zwitterionic, highly dipolar structure of **5 a/b**. The carbon‐selenium coupling constant (only observable in ^77^Se‐enriched **5 b**), ^1^ *J*(^13^C−^77^Se)=199 Hz, indicates significantly increased nucleophilicity of **5 a/b** in comparison to NHCs (^1^ *J*(^13^C−^77^Se)=214–239 Hz),^19^ in line with the elongated carbon‐selenium distance observed in the single‐crystal structure of **5 a** (see above). The chemical shift of the selenium atom (observable both in **5 a** and **b**), *δ*(^77^Se)=258 ppm (vs. (H_3_C)_2_Se as standard), is remarkably higher in comparison to those of standard NHCs (ca. <150 ppm),^19^ suggesting increased π‐acidity and backbonding properties of **5 a/b** compared to common NHCs, in contrast to the finding of an elongated carbon‐selenium bond length in the crystal structure of **5 a** (see above). Applying a correlation of ^77^Se chemical shifts and ^1^ *J*(^13^C−^77^Se) coupling constants with TEP values proposed by Ganter et al.,^19^ TEP=*aδ*(^77^Se)+*b* ^1^ *J*(^13^C−^77^Se)+c with *a*=0.0191, *b*=0.0424, *c*=2040.8, gave for the mesoionic CcC ligand a rather high (unrealistic) value of 2054 cm^−1^ not supported by our experimental data, most likely due to limitations of this unique; however, rather rough correlation (*R* ^2^=0.833) to NHC ligands.^19^ Taken all DFT and experimental results together, CcC is best described as a highly basic, highly nucleophilic, σ‐donating as well as electrophilic, π‐accepting mesoionic metallocarbene.

Cyclic and square voltammetric experiments were conducted to probe for the impact of cobaltoceniumide formation and coordination to the Au(CN)_3_ entity (Figure 4 and Figures S20 to S22 in the Supporting Information) or selenide binding (Figure 5, Figures S23 and S24 in the Supporting Information). In the THF/NBu_4_^+^ PF_6_^−^ electrolyte, **2** exhibits an ideal Nernstian one‐electron redox wave associated with peaks *I*_c_ and *I*_a_ at a half‐wave potential *E*_1/2_ of −1.380 V on the ferrocene/ferrocenium scale (see the Supporting Information, Figures S20 and S21). The latter value is by 48 mV negative of the Co^III/II^ couple CcH^+^/CcH of cobaltocenium hexafluoridophosphate itself (see the Supporting Information, Figure S21). Formal substitution of a proton by an Au(CN)_3_ moiety thus renders the cobaltocenium core slightly more electron rich. On scanning to more negative potential past the Co^III/II^ wave, a second, chemically irreversible reduction, peak II_c_, was detected at a peak potential of −2.475 V (*v*=100 mV s^−1^). Due to the proximity of that peak to the cathodic discharge limit of the solvent, it is associated with a slightly larger peak current than the first, reversible couple. When, after traversing the irreversible reduction peak, the sweep was taken back in the anodic direction, the new peak III_a_ was detected. On repetitive cycling, an associated cathodic counter peak III_c_ (see the Supporting Information, Figure S22) appears such that peaks III_a_/III_c_ constitute a reversible redox couple with a half‐wave potential *E*_1/2_ of −1.715 V. Taken together, our results suggest that the second reduction transforms complex **2** into a considerably more electron‐rich cobaltocenyl gold(I)complex, tentatively assigned as CcAuCN.

**Figure 4:**
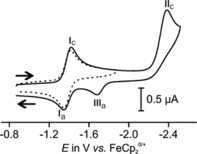
Cyclic voltammogram of **2** (THF, NBu_4_^+^PF_6_^−^ (0.1 m), RT); dotted line: first reduction only; solid line: complete scan.

**Figure 5:**
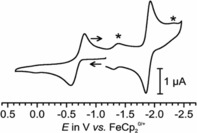
Cyclic voltammogram of **5 a** (THF, NBu_4_^+^PF_6_^−^ (0.1 m), RT); the asterisk marks the redox waves of cobaltocenium impurities in the sample.

In agreement with the much stronger electron‐donating character of the selenido substituent compared to the Au(CN)_3_ moiety, the redox potential of complex **5 a** of −1.882 V is cathodically shifted by as much as 500 mV with respect to the **2**/**2^−^** couple and by 550 mV with respect to the CcH^+^/CcH reduction wave (Figure 5 and Figure S23 in the Supporting Information). The expected, second reduction was not observed and lies clearly outside the accessible solvent window. Complex **5 a** also features an additional quasireversible, anodic wave with significantly increased peak potential splittings (see the Supporting Information, Figure S24) and an *E*_1/2_ of −0.685 V, which has no precedent in other cobaltocenium salts or in **2**. Because the half‐wave potential of this wave falls close to the RSe^−^/RSe couple of aryl‐substituted organoselenides of approximately −0.5 to −0.8 V, we tentatively assign it to the selenium‐centered oxidation of complex **5 a**.^23^

The prominent electronic transition of the purple complexes **5 a** and **b** at 555 nm is most likely associated with charge transfer from the selenide donor to the cobaltocenium‐like acceptor units within this complex. In line with such an interpretation, this band is bleached on oxidation, as well as reduction, giving way to much weaker absorptions at 370 nm (**5 a^+^**) or a similar intense band at 325 nm with a shoulder at 383 nm and the lower‐energy features of a cobaltocene (**5 a^−^**; see Figures S25 and S26 in the Supporting Information).^24^

In summary, cobaltocenylidene (CcC) is a unique mesoionic metallocarbene that forms stable Au^III^ complexes. Structural, spectroscopic, and computational results show that CcC is an especially electron‐rich carbene with electronic properties comparable to cyclic (amino)(alkyl/aryl)carbenes. However, in contrast to these and other N‐heterocyclic carbenes, CcC is significantly more basic, highly polar, and exhibits axial but no peripheral steric shielding in combination with a redox‐responsive cobalt center. Future efforts are directed towards expanding the coordination chemistry of CcC to other transition metals and accessing the CcC ligand by non‐oxidative addition methods, for example, by deprotonation of cobaltocenium salts, aiming at applications of CcC compounds in catalysis and small molecule activation.

CCDC https://www.ccdc.cam.ac.uk/services/structures?id=doi:10.1002/chem.201800147 contain the supplementary crystallographic data for this paper. These data can be obtained free of charge from http://www.ccdc.cam.ac.uk/.

## Conflict of interest

The authors declare no conflict of interest.

## Supporting information

As a service to our authors and readers, this journal provides supporting information supplied by the authors. Such materials are peer reviewed and may be re‐organized for online delivery, but are not copy‐edited or typeset. Technical support issues arising from supporting information (other than missing files) should be addressed to the authors.

Supplementary
