# Supplementary material for: Cobaltocenylidene: A Mesoionic Metalloceno Carbene, Stabilized in a Gold(III) Complex
Source: Chemistry. 2018 Feb 5;24(13):3165–9. doi: 10.1002/chem.201800147 (PMC5888181; doi:10.1002/chem.201800147)
Supplement: Supplementary file 1 — Supplementary [file CHEM-24-3165-s001.pdf]

CHEMISTRY
A European Journal

Supporting Information

Cobaltocenylidene: A Mesoionic Metalloceno Carbene, Stabilized in a Gold(III) Complex

Stefan Vanicek,[a] Maren Podewitz,[a] Christopher Hassenrück,[b] Michael Pittracher,[a] Holger Kopacka,[a] Klaus Wurst,[a] Thomas Müller,[c] Klaus R. Liedl,[a] Rainer F. Winter,[b] and Benno Bildstein*[a]

chem_201800147_sm_miscellaneous_information.pdf

Table of Contents

Experimental Section S2
Electrochemical Section S31
Computational Section S35
References S39

Experimental Section

General Procedures. Synthetic methods, spectroscopic characterization, and single-crystal structure analysis were performed as described recently.[1] Chemicals were obtained commercially and used as received. The starting material cobaltocenium diazonium bis(hexafluoridophosphate) (**1**) was synthesized as recently published.[1]

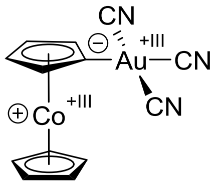

[463,14]
$C_{13}H_9N_3CoAu$

Cobaltoceniumyltricyanidogold(III) (2). A 50 mL round bottom Schlenk flask equipped with a reflux condenser was charged with 20 mL of nitromethane (abs), 0.053 g of AuCN (0.237 mmol, 1.2 equiv.) and 0.031 g KCN (0.474 mmol, 2.4 equiv.) under exclusion of light and under protection from air by an argon atmosphere. After refluxing overnight $K[Au(CN)_2]$ was obtained as a golden solution, accompanied by traces of a brown solid. The reaction mixture was cooled to 0 °C in an ice bath and another 0.015 g of KCN (0.237 mmol, 1.2 equiv.) was added. After 5 min of continuous stirring at 0 °C 0.100 g of **1** (0.198 mmol, 1 equiv.) was added and the reaction mixture concomitantly turned amber. The temperature was held for 3 h until the mixture was allowed to warm to room temperature and the reaction was completed by stirring overnight. The solvent was removed *in vacuo* and a ^{1}H-NMR was performed as reaction control (see Figure S2), indicating a conversion of 97.3 %, whereby the byproduct formed represented nitromethylcobaltocenium hexafluoridophosphate. _Note 1:_ Any attempt to vary the parameters, such as the solvent (e.g. nitroethane or 2-nitropropane), the stoichiometry or the temperature program, lead to significantly inferior results. Dichloromethane and small amounts of water were added and the product was extracted with CH_2Cl_2. The aqueous phase was removed and the organic layer was extracted one more time with a small amount of water. The organic phase was dried with sodium sulfate, the drying agent was filtered off and the solvent was removed on a rotary evaporator. 5 mL of acetone was added, dissolving the reddish byproduct nitromethylcobaltocenium hexafluoridophosphate with the aid of ultrasound. The procedure was iterated three times until **2** was obtained as a crystalline ochre powder. _Note 2:_ Any other work-up procedure (e.g. chromatography, RPC, precipitation, recrystallization from acetone or water, etc.) caused inferior results concerning yield or purity, respectively. Drying *in vacuo* afforded 0.044 g (0.095 mmol) of **2**, representing a yield of 48 %. Compound **2** is highly air-, thermo- and water stable. It is soluble in dichloromethane, acetonitrile, methanol, nitromethane, dimethylsulfoxide and water. ^{1}H-NMR (300 MHz, CD_3CN): δ 5.67 (s, 5H, Cp), 5.70 (pseudo-t, 2H, *J* = 2.0 Hz, C3/C4 of substituted Cp), 5.75 (pseudo-t, 2H, *J* = 2.0 Hz, C2/C5 of substituted Cp) ppm. ^{13}C-NMR (75 MHz, CD_3CN): δ 86.5 (Cp), 86.7 (C3/C4 of substituted Cp), 93.3 (C2/C5 of substituted Cp), 114.2 (carbene carbon), 120.7 (*cis*-cyanides) ppm; the resonance signal of the *trans*-cyanide carbon was not observed. ^{1}H-NMR (300 MHz, DMSO): δ 5.67 (pseudo-t, 2H, *J* = 2.0 Hz, C3/C4 of substituted Cp), 5.81 (s, 5H, Cp), 5.86 (pseudo-t, 2H, *J* = 2.0 Hz, C2/C5 of substituted Cp) ppm. _Note 3:_ The chemical shifts of the pseudo-triplets are highly solvent depending. ^{13}C-NMR (75 MHz, DMSO): δ 85.3 (C3/C4 of substituted Cp), 85.4 (Cp), 91.7 (C2/C5 of substituted Cp), 113.6 (carbene carbon), 120.4 (*cis*-cyanides), 123.7 (*trans*-cyanide) ppm. _Note 4:_ Cyanide chemical shifts were in compliance with literature values.[2] MS (ESI pos, [m/z]): 463.99 $[M+H]^+$. IR (ATR): 3112 (ν_{C-H}), 2204 ($\nu_{C\equiv N}$), 2172 ($\nu_{C\equiv N}$), 1412 ($\nu_{C=C}$), 1383, 1340, 1051, 1024, 1005, 870 ($\delta_{op(C-H)}$), 504, 466 ($\nu_{as(Co-ring)}$), 440 ($\nu_{as(Co-ring)}$) cm^{-1}. **2** crystallizes readily out of acetonitrile as large crystals of millimeter dimensions that lend themselves for X-ray crystallographc structure determination, mp: >300 °C (dec).

Spectra

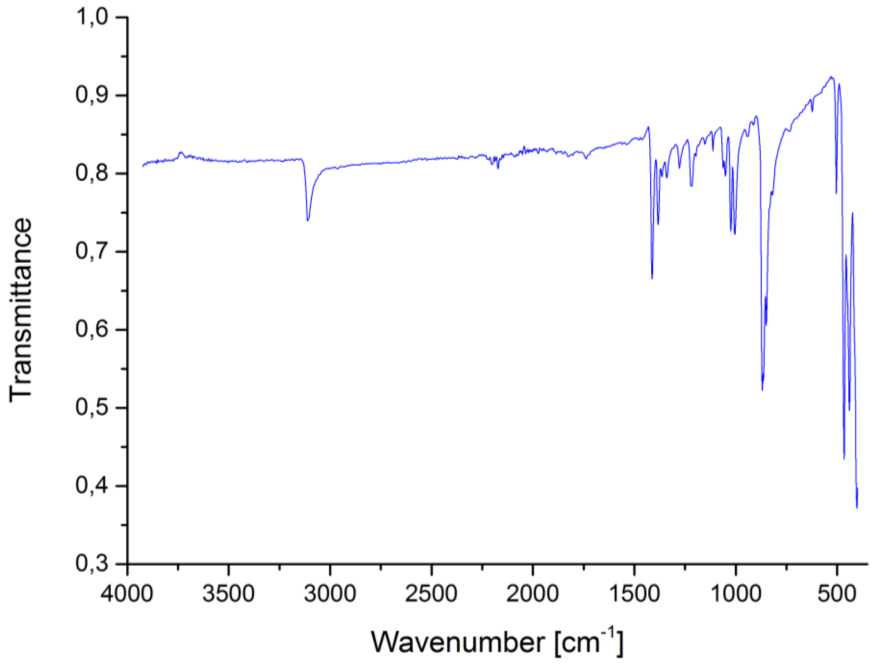

Figure S1. IR-spectrum (ATR, [cm^{-1}]) of **2**.

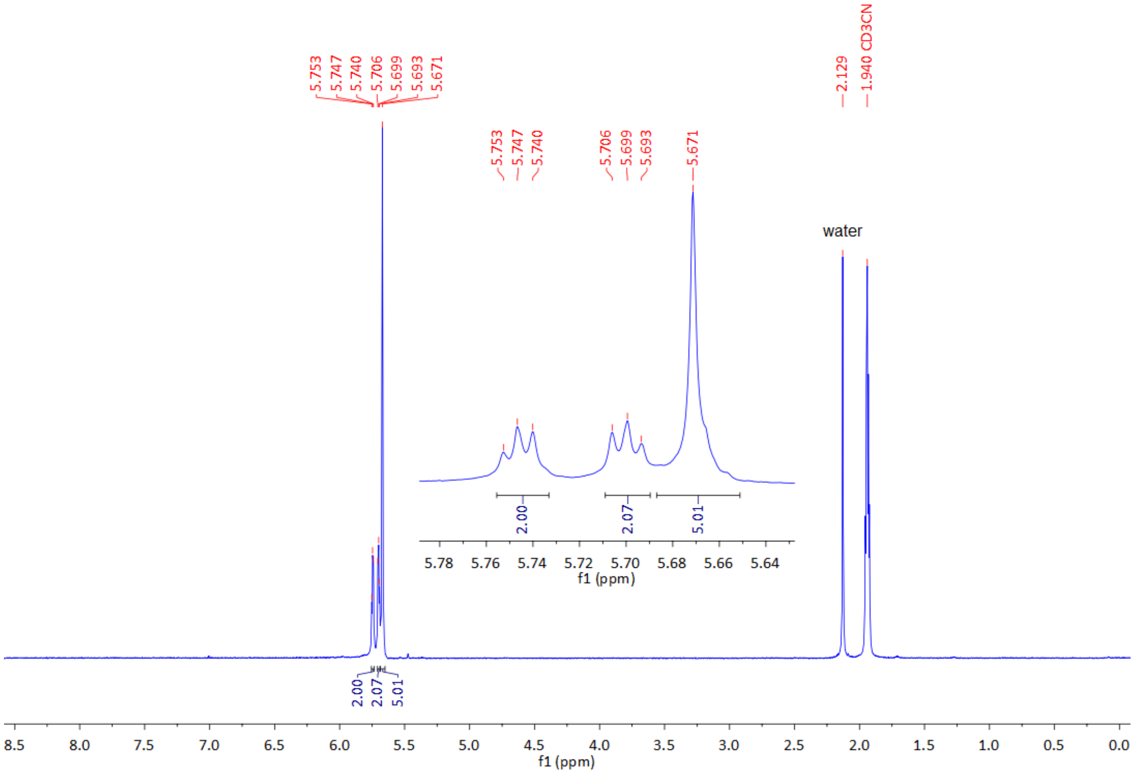

Figure S2. ^{1}H-NMR (300 MHz, CD_3CN, [ppm]) of **2**.

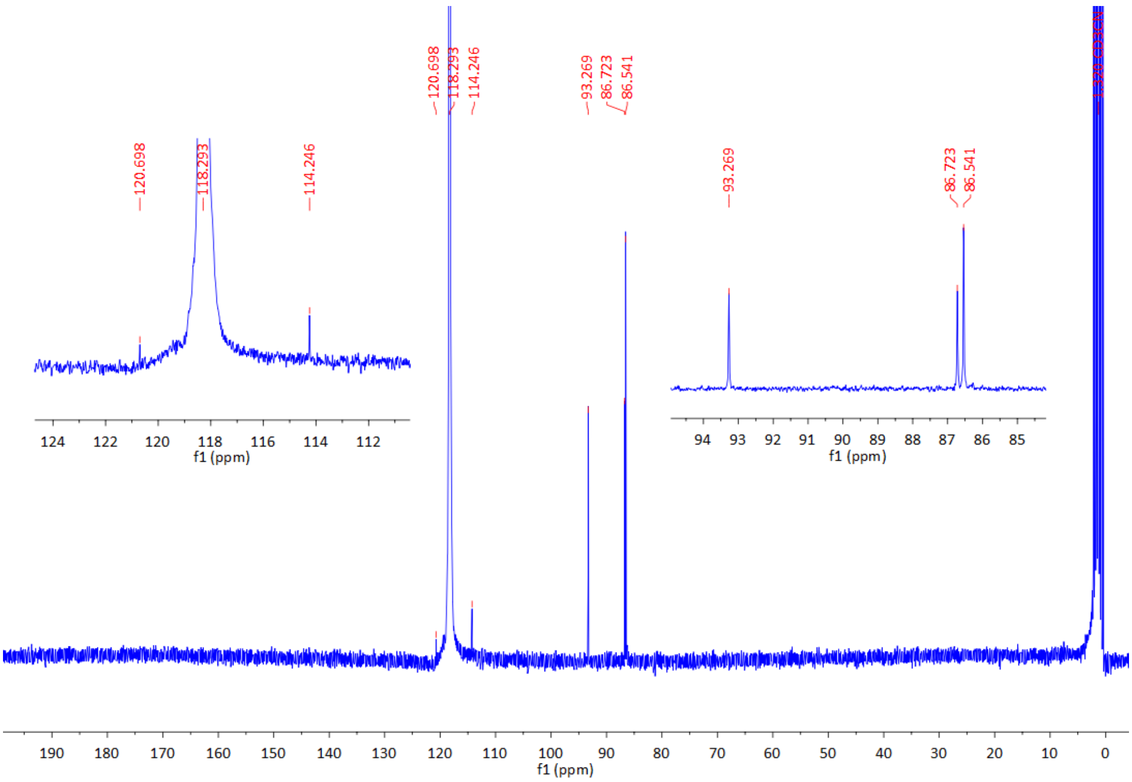

Figure S3. ^{13}C-NMR (75 MHz, CD_3CN, [ppm]) of **2**.

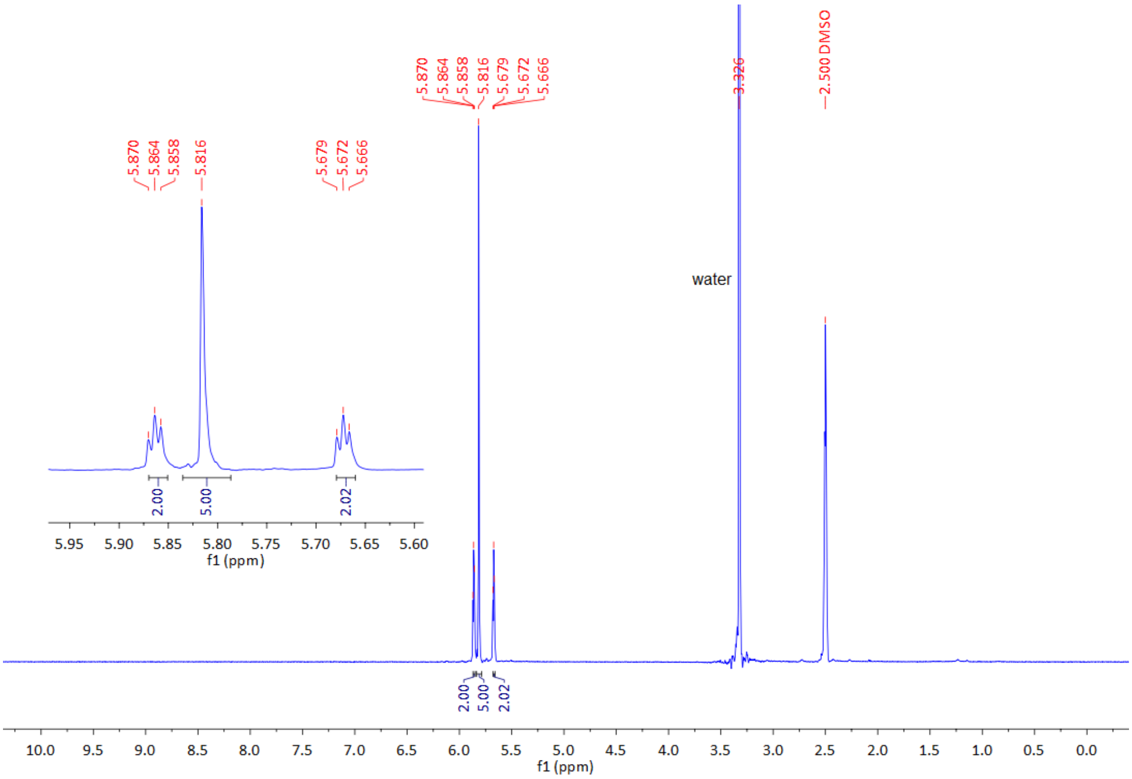

Figure S4. ^{1}H-NMR (300 MHz, DMSO, [ppm]) of **2**.

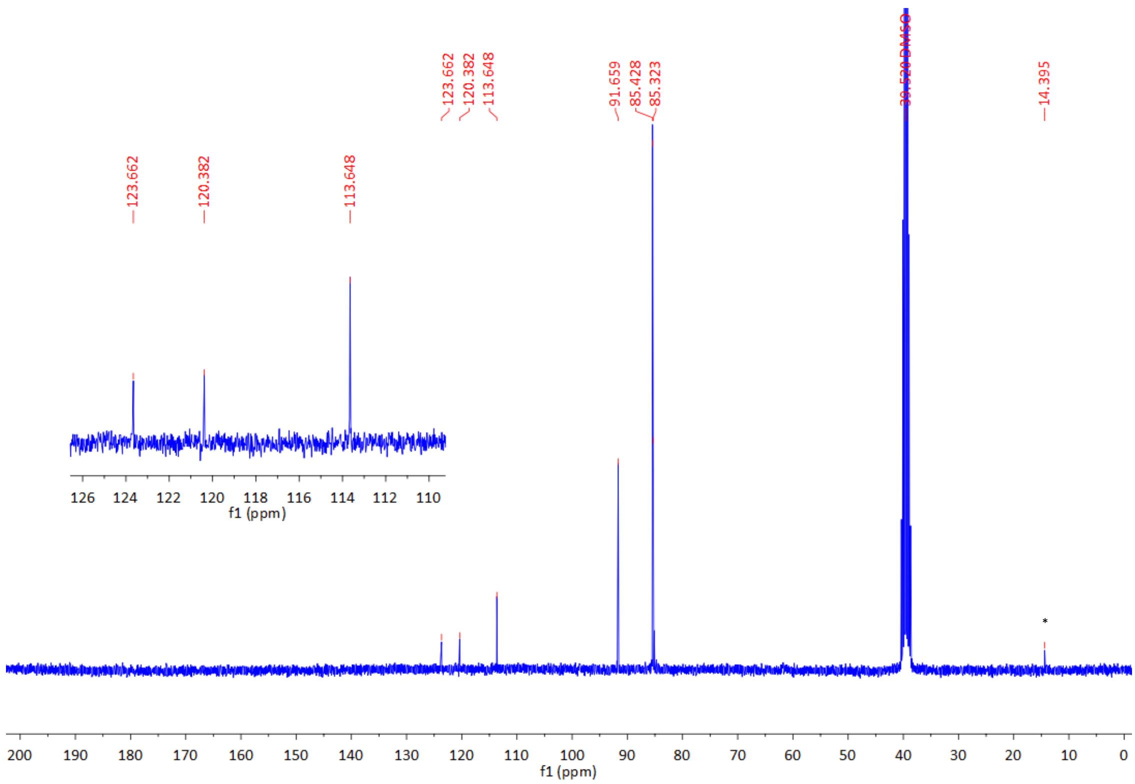

Figure S5. ^{13}C-NMR (75 MHz, DMSO, [ppm]) of **2** (* represents impurity of the solvent).

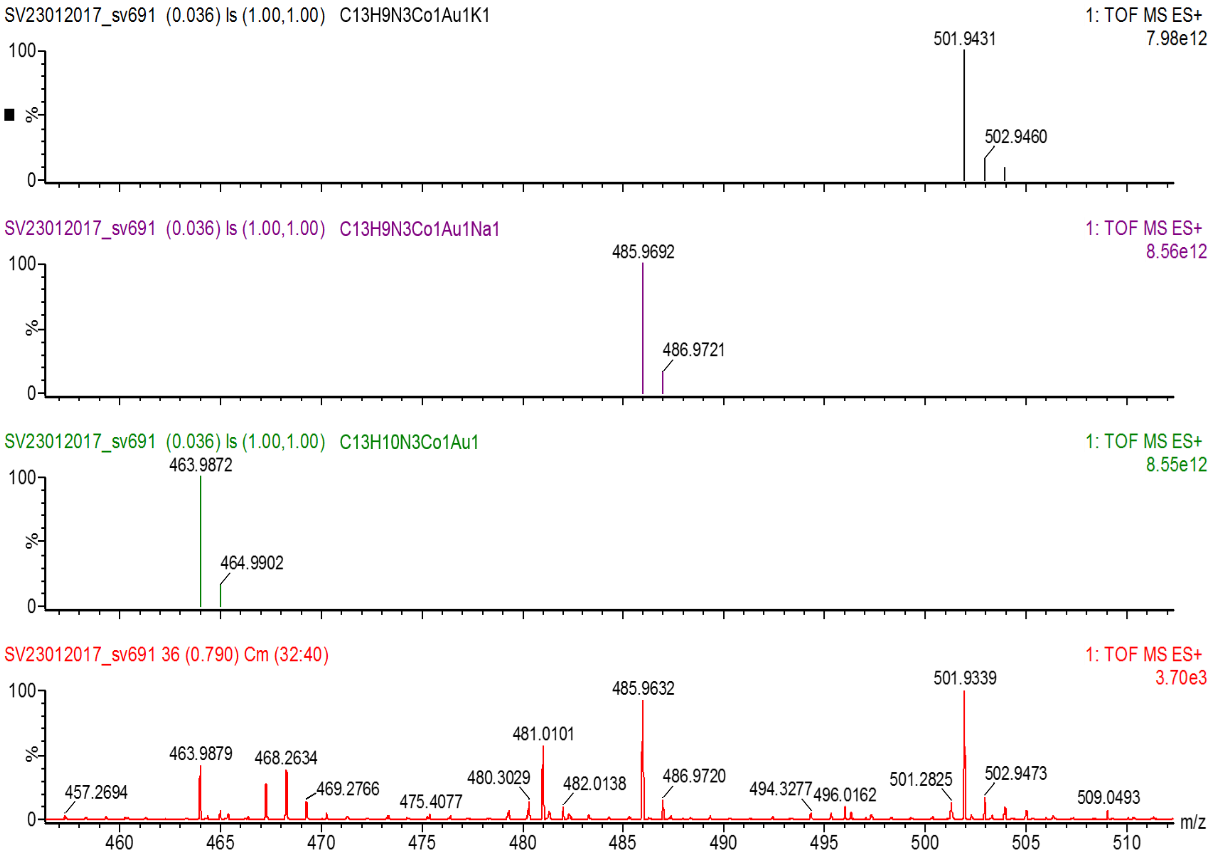

Figure S6. MS (ESI pos, [m/z]; *bottom*: experimental, *simulated*: simulated) of **2**.

Crystallographic data

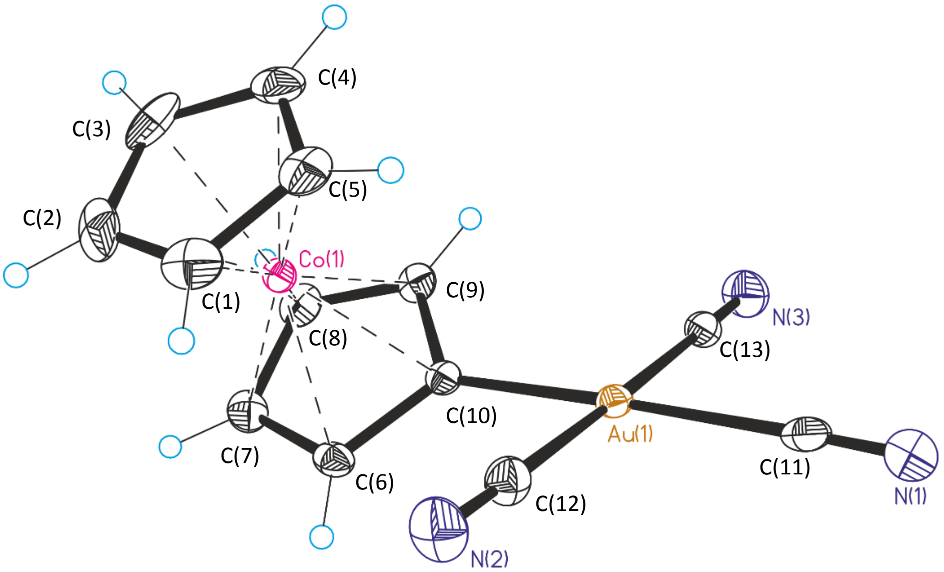

Figure S7. X-ray single crystal structure analysis of **2**.

Table 1. Crystal data and structure refinement for **2**.

Identification code	bi88	
Empirical formula	$C_{13}H_9AuCoN_3$	
Formula weight	463.13	
Temperature	203(2) K	
Wavelength	0.71073 Å	
Crystal system	Triclinic	
Space group	P $\bar{1}$ (no. 2)	
Unit cell dimensions	*a* = 7.3652(9) Å	*α* = 102.739(5)°
	b = 8.6232(12) Å	*ß* = 94.242(5)°
	c = 10.2546(14) Å	*γ* = 95.111(5)°
Volume	629.79(15) $Å^3$	
Z	2	
Density (calculated)	2.442 Mg/m^3	
Absorption coefficient	12.933 mm^{-1}	
F(000)	428	
Crystal size	0.180 x 0.160 x 0.120 mm^3	
Theta range for data collection	2.436 to 27.000°	
Index ranges	-9<*h*<9, -11<*k*<11, -13<*l*<13	
Reflections collected	18168	
Independent reflections	2737 [R(int) = 0.0421]	
Completeness to theta = 25.242°	100.0 %	

Absorption correction	Semi-empirical from equivalents
Max. and min. transmission	0.342 and 0.202
Refinement method	Full-matrix least-squares on F^2
Data / restraints / parameters	2737 / 0 / 164
Goodness-of-fit on F^2	1.140
Final *R* indices [I>2sigma(I)]	*R*1 = 0.0172, w*R*2 = 0.0414
R indices (all data)	*R*1 = 0.0186, w*R*2 = 0.0419
Extinction coefficient	0.0072(3)
Largest diff. peak and hole	0.796 and -0.735 e.Å^{-3}

Table 2. Atomic coordinates (x 10^4) and equivalent isotropic displacement parameters (Å^2 x 10^3) for **2**. U(eq) is defined as one third of the trace of the orthogonalized U^{ij} tensor.

	x	y	z	U(eq)
Au(1)	2801(1)	4794(1)	5937(1)	29(1)
Co(1)	2756(1)	1784(1)	7955(1)	25(1)
N(1)	1328(6)	7635(5)	4853(4)	60(1)
N(2)	2669(6)	6671(5)	8912(4)	58(1)
N(3)	2982(6)	2791(5)	3019(4)	55(1)
C(1)	1424(6)	2879(6)	9509(5)	52(1)
C(2)	1979(7)	1448(7)	9730(5)	62(1)
C(3)	1156(7)	199(6)	8672(5)	58(1)
C(4)	91(5)	906(5)	7796(4)	48(1)
C(5)	279(5)	2535(5)	8319(5)	45(1)
C(6)	5059(5)	3202(5)	7839(4)	34(1)
C(7)	5500(5)	1683(5)	7987(4)	40(1)
C(8)	4565(5)	521(5)	6888(4)	38(1)
C(9)	3554(5)	1330(4)	6068(3)	33(1)
C(10)	3862(4)	3003(4)	6648(3)	27(1)
C(11)	1815(5)	6654(5)	5203(4)	39(1)
C(12)	2719(6)	6010(5)	7833(4)	40(1)
C(13)	2902(5)	3516(5)	4052(4)	35(1)

Table 3. Bond lengths [Å] and angles [°] for **2**.

Au(1)-C(12)	2.003(4)	C(2)-Co(1)-C(8)	124.47(19)
Au(1)-C(13)	2.013(4)	C(3)-Co(1)-C(8)	106.45(18)
Au(1)-C(10)	2.038(3)	C(4)-Co(1)-C(8)	120.49(17)
Au(1)-C(11)	2.080(4)	C(5)-Co(1)-C(8)	155.74(18)
Co(1)-C(2)	2.020(4)	C(2)-Co(1)-C(6)	121.96(19)
Co(1)-C(3)	2.021(4)	C(3)-Co(1)-C(6)	156.6(2)
Co(1)-C(4)	2.021(4)	C(4)-Co(1)-C(6)	161.21(17)
Co(1)-C(5)	2.023(4)	C(5)-Co(1)-C(6)	125.69(16)
Co(1)-C(8)	2.026(3)	C(8)-Co(1)-C(6)	68.68(15)
Co(1)-C(6)	2.028(3)	C(2)-Co(1)-C(7)	107.86(18)
Co(1)-C(7)	2.029(4)	C(3)-Co(1)-C(7)	120.57(19)
Co(1)-C(9)	2.029(3)	C(4)-Co(1)-C(7)	156.26(18)
Co(1)-C(1)	2.032(4)	C(5)-Co(1)-C(7)	162.35(18)
Co(1)-C(10)	2.048(3)	C(8)-Co(1)-C(7)	40.95(16)
N(1)-C(11)	1.069(5)	C(6)-Co(1)-C(7)	40.83(15)
N(2)-C(12)	1.132(5)	C(2)-Co(1)-C(9)	161.0(2)
N(3)-C(13)	1.114(5)	C(3)-Co(1)-C(9)	123.62(19)
C(1)-C(5)	1.390(6)	C(4)-Co(1)-C(9)	106.75(17)
C(1)-C(2)	1.393(7)	C(5)-Co(1)-C(9)	121.02(17)
C(1)-H(1)	0.9500	C(8)-Co(1)-C(9)	40.88(15)
C(2)-C(3)	1.405(8)	C(6)-Co(1)-C(9)	68.57(15)
C(2)-H(2)	0.9500	C(7)-Co(1)-C(9)	68.83(16)
C(3)-C(4)	1.420(7)	C(2)-Co(1)-C(1)	40.2(2)
C(3)-H(3)	0.9500	C(3)-Co(1)-C(1)	68.00(19)
C(4)-C(5)	1.379(6)	C(4)-Co(1)-C(1)	67.51(18)
C(4)-H(4)	0.9500	C(5)-Co(1)-C(1)	40.09(19)
C(5)-H(5)	0.9500	C(8)-Co(1)-C(1)	161.86(19)
C(6)-C(7)	1.415(5)	C(6)-Co(1)-C(1)	109.12(17)
C(6)-C(10)	1.421(5)	C(7)-Co(1)-C(1)	125.65(18)
C(6)-H(6)	0.9500	C(9)-Co(1)-C(1)	156.60(18)
C(7)-C(8)	1.419(6)	C(2)-Co(1)-C(10)	157.0(2)
C(7)-H(7)	0.9500	C(3)-Co(1)-C(10)	160.8(2)
C(8)-C(9)	1.416(5)	C(4)-Co(1)-C(10)	123.88(16)
C(8)-H(8)	0.9500	C(5)-Co(1)-C(10)	108.00(16)
C(9)-C(10)	1.425(5)	C(8)-Co(1)-C(10)	68.95(14)
C(9)-H(9)	0.9500	C(6)-Co(1)-C(10)	40.81(14)
C(12)-Au(1)-C(13)	178.34(14)	C(7)-Co(1)-C(10)	68.96(14)
C(12)-Au(1)-C(10)	89.26(14)	C(9)-Co(1)-C(10)	40.91(14)
C(13)-Au(1)-C(10)	89.10(14)	C(1)-Co(1)-C(10)	121.98(17)
C(12)-Au(1)-C(11)	90.99(15)	C(5)-C(1)-C(2)	108.1(4)
C(13)-Au(1)-C(11)	90.66(16)	C(5)-C(1)-Co(1)	69.6(2)
C(10)-Au(1)-C(11)	177.92(13)	C(2)-C(1)-Co(1)	69.4(2)
C(2)-Co(1)-C(3)	40.7(2)	C(5)-C(1)-H(1)	125.9
C(2)-Co(1)-C(4)	68.3(2)	C(2)-C(1)-H(1)	125.9
C(3)-Co(1)-C(4)	41.1(2)	Co(1)-C(1)-H(1)	126.6
C(2)-Co(1)-C(5)	67.75(19)	C(1)-C(2)-C(3)	108.2(4)
C(3)-Co(1)-C(5)	68.09(18)	C(1)-C(2)-Co(1)	70.3(2)
C(4)-Co(1)-C(5)	39.87(18)	C(3)-C(2)-Co(1)	69.7(2)

C(1)-C(2)-H(2)	125.9	C(6)-C(7)-C(8)	107.6(3)
C(3)-C(2)-H(2)	125.9	C(6)-C(7)-Co(1)	69.5(2)
Co(1)-C(2)-H(2)	125.6	C(8)-C(7)-Co(1)	69.4(2)
C(2)-C(3)-C(4)	106.9(4)	C(6)-C(7)-H(7)	126.2
C(2)-C(3)-Co(1)	69.6(3)	C(8)-C(7)-H(7)	126.2
C(4)-C(3)-Co(1)	69.5(2)	Co(1)-C(7)-H(7)	126.4
C(2)-C(3)-H(3)	126.6	C(9)-C(8)-C(7)	108.0(3)
C(4)-C(3)-H(3)	126.6	C(9)-C(8)-Co(1)	69.67(19)
Co(1)-C(3)-H(3)	125.9	C(7)-C(8)-Co(1)	69.6(2)
C(5)-C(4)-C(3)	108.0(4)	C(9)-C(8)-H(8)	126.0
C(5)-C(4)-Co(1)	70.1(2)	C(7)-C(8)-H(8)	126.0
C(3)-C(4)-Co(1)	69.4(2)	Co(1)-C(8)-H(8)	126.3
C(5)-C(4)-H(4)	126.0	C(8)-C(9)-C(10)	108.6(3)
C(3)-C(4)-H(4)	126.0	C(8)-C(9)-Co(1)	69.5(2)
Co(1)-C(4)-H(4)	126.0	C(10)-C(9)-Co(1)	70.26(19)
C(4)-C(5)-C(1)	108.8(4)	C(8)-C(9)-H(9)	125.7
C(4)-C(5)-Co(1)	70.0(2)	C(10)-C(9)-H(9)	125.7
C(1)-C(5)-Co(1)	70.3(2)	Co(1)-C(9)-H(9)	126.1
C(4)-C(5)-H(5)	125.6	C(6)-C(10)-C(9)	106.8(3)
C(1)-C(5)-H(5)	125.6	C(6)-C(10)-Au(1)	125.8(3)
Co(1)-C(5)-H(5)	125.7	C(9)-C(10)-Au(1)	127.4(3)
C(7)-C(6)-C(10)	109.0(3)	C(6)-C(10)-Co(1)	68.82(19)
C(7)-C(6)-Co(1)	69.6(2)	C(9)-C(10)-Co(1)	68.83(19)
C(10)-C(6)-Co(1)	70.37(19)	Au(1)-C(10)-Co(1)	127.08(16)
C(7)-C(6)-H(6)	125.5	N(1)-C(11)-Au(1)	178.3(4)
C(10)-C(6)-H(6)	125.5	N(2)-C(12)-Au(1)	178.7(4)
Co(1)-C(6)-H(6)	126.1	N(3)-C(13)-Au(1)	178.6(4)

Table 4. Anisotropic displacement parameters (Å^2 x 10^3) for **2**. The anisotropic displacement factor exponent takes the form: $-2\pi^2[h^2 a^{*2}U^{11} + ... + 2 h k a^* b^* U^{12}]$

	U^{11}	U^{22}	U^{33}	U^{23}	U^{13}	U^{12}
Au(1)	28(1)	30(1)	27(1)	6(1)	3(1)	-4(1)
Co(1)	23(1)	32(1)	20(1)	4(1)	5(1)	2(1)
N(1)	58(3)	58(3)	61(3)	18(2)	-9(2)	-4(2)
N(2)	79(3)	48(2)	42(2)	-3(2)	6(2)	17(2)
N(3)	59(2)	67(3)	36(2)	10(2)	6(2)	-2(2)
C(1)	49(2)	59(3)	43(2)	-7(2)	25(2)	0(2)
C(2)	52(3)	112(4)	35(2)	32(3)	20(2)	15(3)
C(3)	65(3)	49(2)	77(3)	31(2)	47(3)	11(2)
C(4)	32(2)	60(3)	48(2)	6(2)	12(2)	-11(2)
C(5)	28(2)	56(2)	57(3)	19(2)	22(2)	10(2)
C(6)	25(2)	42(2)	31(2)	4(2)	4(1)	-2(1)
C(7)	25(2)	55(2)	44(2)	17(2)	6(2)	9(2)
C(8)	39(2)	38(2)	40(2)	7(2)	20(2)	12(2)
C(9)	40(2)	35(2)	24(2)	2(1)	12(1)	3(1)
C(10)	24(2)	34(2)	24(2)	7(1)	8(1)	1(1)
C(11)	34(2)	37(2)	41(2)	4(2)	1(2)	-11(2)
C(12)	47(2)	36(2)	39(2)	8(2)	5(2)	7(2)
C(13)	32(2)	44(2)	29(2)	12(2)	2(1)	-4(1)

Table 5. Hydrogen coordinates (x 10^4) and isotropic displacement parameters ($Å^2$x 10^{3}) for **2**.

	x	y	z	U(eq)
H(1)	1768	3911	10074	63
H(2)	2776	1335	10467	75
H(3)	1286	-907	8564	70
H(4)	-624	355	6992	58
H(5)	-283	3298	7930	54
H(6)	5493	4195	8438	40
H(7)	6282	1478	8695	48
H(8)	4609	-602	6730	46
H(9)	2798	839	5266	40

Table 6. Torsion angles [°] for **2**.

C(5)-C(1)-C(2)-C(3)	-0.6(5)	C(6)-C(7)-C(8)-Co(1)	-59.3(2)
Co(1)-C(1)-C(2)-C(3)	-59.6(3)	C(7)-C(8)-C(9)-C(10)	0.3(4)
C(5)-C(1)-C(2)-Co(1)	59.1(3)	Co(1)-C(8)-C(9)-C(10)	59.6(2)
C(1)-C(2)-C(3)-C(4)	0.3(5)	C(7)-C(8)-C(9)-Co(1)	-59.3(2)
Co(1)-C(2)-C(3)-C(4)	-59.7(3)	C(7)-C(6)-C(10)-C(9)	0.6(4)
C(1)-C(2)-C(3)-Co(1)	60.1(3)	Co(1)-C(6)-C(10)-C(9)	-58.5(2)
C(2)-C(3)-C(4)-C(5)	0.1(4)	C(7)-C(6)-C(10)-Au(1)	-179.7(2)
Co(1)-C(3)-C(4)-C(5)	-59.8(3)	Co(1)-C(6)-C(10)-Au(1)	121.2(2)
C(2)-C(3)-C(4)-Co(1)	59.8(3)	C(7)-C(6)-C(10)-Co(1)	59.1(2)
C(3)-C(4)-C(5)-C(1)	-0.4(4)	C(8)-C(9)-C(10)-C(6)	-0.6(4)
Co(1)-C(4)-C(5)-C(1)	-59.8(3)	Co(1)-C(9)-C(10)-C(6)	58.5(2)
C(3)-C(4)-C(5)-Co(1)	59.3(3)	C(8)-C(9)-C(10)-Au(1)	179.7(2)
C(2)-C(1)-C(5)-C(4)	0.6(5)	Co(1)-C(9)-C(10)-Au(1)	-121.2(3)
Co(1)-C(1)-C(5)-C(4)	59.6(3)	C(8)-C(9)-C(10)-Co(1)	-59.1(2)
C(2)-C(1)-C(5)-Co(1)	-58.9(3)		
C(10)-C(6)-C(7)-C(8)	-0.4(4)		
Co(1)-C(6)-C(7)-C(8)	59.2(2)		
C(10)-C(6)-C(7)-Co(1)	-59.6(2)		
C(6)-C(7)-C(8)-C(9)	0.0(4)		
Co(1)-C(7)-C(8)-C(9)	59.3(2)		

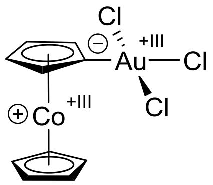

[491.44]
$C_{10}H_9CoAuCl_3$

Trichloridocobaltoceniumylgold(III) (3). A Schlenk flask was charged with 5 mL of nitromethane (abs), 0.050 g of **1** (0.099 mmol, 1 equiv.), 0.049 g of $(PPh_3)AuCl$ (0.099 mmol, 1 equiv.) and 0.014 g of KCl (0.198 mmol, 2 equiv.). The reaction mixture was stirred at room temperature overnight. The solvent was removed on a rotary evaporator and the yellow product was dissolved in small amounts of cold methanol, thus separating white solid byproduct. **3** was crystallized by diffusion of pentane into a solution of the complex in methanol. *Note 5:* No satisfactory work up procedure (e.g. chromatography, precipitation, recrystallization out of various solvent combinations, etc.) could be found for this derivative. The solids obtained still contained triphenylphosphine and inorganic salts as impurities. Subsequently, neither utilization of photochemistry according to literature,[3] nor replacement of the chlorido(triphenylphosphine)gold(I) educt with $Cs[AuCl_2]$ lead to significant improvement, in the latter inter alia based on the inferior stability of $Cs[AuCl_2]$ compared to its cyanide congener.

Crystallographic data

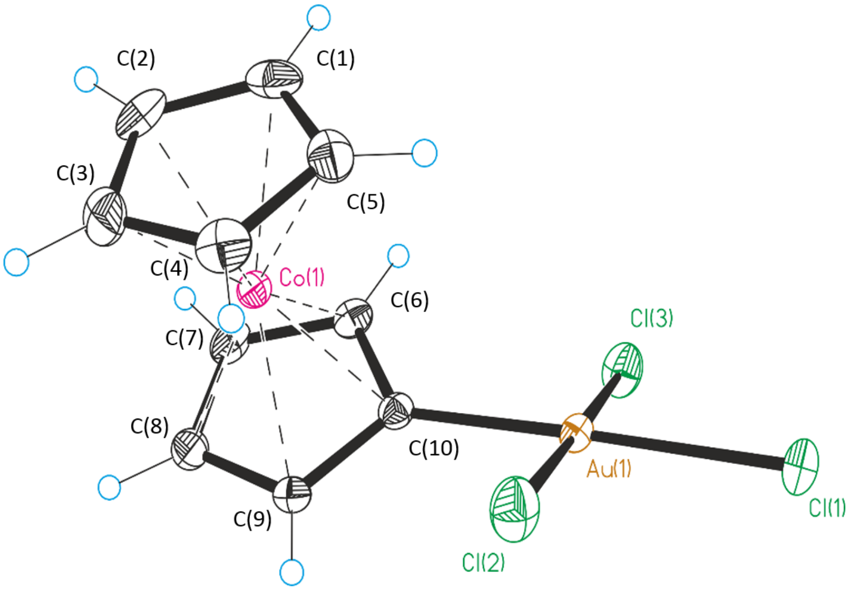

Figure S8. X-ray single crystal structure analysis of **3**.

Table 7. Crystal data and structure refinement for **3**.

Identification code	bi64	
Empirical formula	$C_{10}H_9AuCl_3Co$	
Formula weight	491.42	
Temperature	193(2) K	
Wavelength	0.71073 Å	
Crystal system	Monoclinic	
Space group	$P2_1/n$ (no. 14)	
Unit cell dimensions	*a* = 8.8278(4) Å	*α* = 90°
	b = 10.3122(4) Å	*ß* = 90.946(1)°
	c = 13.1168(6) Å	*γ* = 90°
Volume	1193.91(9) $Å^3$	
Z	4	
Density (calculated)	2.734 Mg/m^3	
Absorption coefficient	14.294 mm^{-1}	
F(000)	904	
Crystal size	0.150 x 0.110 x 0.080 mm^3	
Theta range for data collection	2.513 to 26.097°.	
Index ranges	$-10<h<10$, $-12<k<12$, $-15<l<16$	
Reflections collected	26093	
Independent reflections	2372 [*R*(int) = 0.0296]	
Completeness to theta = 25.242°	100.0 %	
Refinement method	Full-matrix least-squares on F^2	
Data / restraints / parameters	2372 / 0 / 137	
Goodness-of-fit on F^2	1.119	
Final R indices [*I*>2sigma(I)]	*R*1 = 0.0152, w*R*2 = 0.0370	
R indices (all data)	*R*1 = 0.0163, w*R*2 = 0.0375	
Extinction coefficient	0.00208(10)	
Largest diff. peak and hole	0.959 and -0.602 e.$Å^{-3}$	

Table 8. Atomic coordinates (x 10^4) and equivalent isotropic displacement parameters ($Å^2$ x 10^3) for **3**. U(eq) is defined as one third of the trace of the orthogonalized U^{ij} tensor.

	x	y	z	U(eq)
Au(1)	5906(1)	3481(1)	6274(1)	25(1)
Co(1)	2230(1)	1925(1)	5869(1)	24(1)
Cl(1)	7579(1)	5096(1)	6876(1)	37(1)
Cl(2)	5002(1)	3191(1)	7891(1)	47(1)
Cl(3)	6771(1)	3722(1)	4651(1)	44(1)
C(1)	779(4)	3383(4)	5518(3)	43(1)
C(2)	-13(4)	2201(4)	5587(3)	44(1)
C(3)	213(4)	1705(4)	6577(3)	44(1)
C(4)	1138(4)	2582(4)	7119(3)	40(1)
C(5)	1492(4)	3619(3)	6463(3)	38(1)
C(6)	3816(4)	1979(3)	4774(2)	30(1)
C(7)	2994(4)	792(3)	4718(3)	35(1)
C(8)	3190(4)	145(3)	5667(3)	35(1)
C(9)	4121(4)	927(3)	6308(3)	30(1)
C(10)	4530(3)	2062(3)	5753(2)	25(1)

Table 9. Bond lengths [Å] and angles [°] for **3**.

Au(1)-C(10)	2.014(3)	C(10)-Au(1)-Cl(3)	88.42(9)
Au(1)-Cl(3)	2.2874(9)	C(10)-Au(1)-Cl(2)	90.10(9)
Au(1)-Cl(2)	2.2988(10)	Cl(3)-Au(1)-Cl(2)	178.51(3)
Au(1)-Cl(1)	2.3536(8)	C(10)-Au(1)-Cl(1)	178.24(9)
Co(1)-C(6)	2.023(3)	Cl(3)-Au(1)-Cl(1)	91.06(3)
Co(1)-C(1)	2.024(3)	Cl(2)-Au(1)-Cl(1)	90.42(3)
Co(1)-C(5)	2.025(3)	C(6)-Co(1)-C(1)	105.11(15)
Co(1)-C(2)	2.028(4)	C(6)-Co(1)-C(5)	118.71(15)
Co(1)-C(7)	2.032(3)	C(1)-Co(1)-C(5)	40.55(17)
Co(1)-C(4)	2.032(4)	C(6)-Co(1)-C(2)	123.45(15)
Co(1)-C(3)	2.035(4)	C(1)-Co(1)-C(2)	40.70(17)
Co(1)-C(9)	2.036(3)	C(5)-Co(1)-C(2)	68.29(17)
Co(1)-C(8)	2.040(3)	C(6)-Co(1)-C(7)	41.09(14)
Co(1)-C(10)	2.044(3)	C(1)-Co(1)-C(7)	118.34(16)
C(1)-C(5)	1.403(6)	C(5)-Co(1)-C(7)	153.44(16)
C(1)-C(2)	1.409(6)	C(2)-Co(1)-C(7)	106.27(15)
C(1)-H(1)	0.9500	C(6)-Co(1)-C(4)	155.08(15)
C(2)-C(3)	1.407(6)	C(1)-Co(1)-C(4)	68.22(16)
C(2)-H(2)	0.9500	C(5)-Co(1)-C(4)	40.70(16)
C(3)-C(4)	1.404(6)	C(2)-Co(1)-C(4)	68.03(16)
C(3)-H(3)	0.9500	C(7)-Co(1)-C(4)	163.44(16)
C(4)-C(5)	1.411(5)	C(6)-Co(1)-C(3)	161.48(16)
C(4)-H(4)	0.9500	C(1)-Co(1)-C(3)	68.34(16)
C(5)-H(5)	0.9500	C(5)-Co(1)-C(3)	68.33(16)
C(6)-C(7)	1.423(5)	C(2)-Co(1)-C(3)	40.51(17)
C(6)-C(10)	1.425(4)	C(7)-Co(1)-C(3)	125.37(16)
C(6)-H(6)	0.9500	C(4)-Co(1)-C(3)	40.39(17)
C(7)-C(8)	1.420(5)	C(6)-Co(1)-C(9)	68.95(14)
C(7)-H(7)	0.9500	C(1)-Co(1)-C(9)	162.34(16)
C(8)-C(9)	1.417(5)	C(5)-Co(1)-C(9)	126.48(16)
C(8)-H(8)	0.9500	C(2)-Co(1)-C(9)	156.34(16)
C(9)-C(10)	1.427(5)	C(7)-Co(1)-C(9)	68.82(14)
C(9)-H(9)	0.9500	C(4)-Co(1)-C(9)	109.76(15)
		C(3)-Co(1)-C(9)	122.33(15)

C(6)-Co(1)-C(8)	68.84(14)	C(3)-C(4)-Co(1)	69.9(2)
C(1)-Co(1)-C(8)	154.36(16)	C(5)-C(4)-Co(1)	69.4(2)
C(5)-Co(1)-C(8)	164.31(16)	C(3)-C(4)-H(4)	125.9
C(2)-Co(1)-C(8)	120.58(16)	C(5)-C(4)-H(4)	125.9
C(7)-Co(1)-C(8)	40.82(14)	Co(1)-C(4)-H(4)	126.4
C(4)-Co(1)-C(8)	127.45(15)	C(1)-C(5)-C(4)	107.9(3)
C(3)-Co(1)-C(8)	109.13(15)	C(1)-C(5)-Co(1)	69.7(2)
C(9)-Co(1)-C(8)	40.69(13)	C(4)-C(5)-Co(1)	69.9(2)
C(6)-Co(1)-C(10)	41.01(13)	C(1)-C(5)-H(5)	126.1
C(1)-Co(1)-C(10)	123.90(15)	C(4)-C(5)-H(5)	126.1
C(5)-Co(1)-C(10)	107.17(14)	Co(1)-C(5)-H(5)	125.9
C(2)-Co(1)-C(10)	160.89(15)	C(7)-C(6)-C(10)	108.4(3)
C(7)-Co(1)-C(10)	69.03(13)	C(7)-C(6)-Co(1)	69.80(19)
C(4)-Co(1)-C(10)	121.47(14)	C(10)-C(6)-Co(1)	70.26(18)
C(3)-Co(1)-C(10)	156.91(16)	C(7)-C(6)-H(6)	125.8
C(9)-Co(1)-C(10)	40.95(13)	C(10)-C(6)-H(6)	125.8
C(8)-Co(1)-C(10)	68.73(13)	Co(1)-C(6)-H(6)	125.7
C(5)-C(1)-C(2)	108.0(3)	C(8)-C(7)-C(6)	107.8(3)
C(5)-C(1)-Co(1)	69.8(2)	C(8)-C(7)-Co(1)	69.89(19)
C(2)-C(1)-Co(1)	69.8(2)	C(6)-C(7)-Co(1)	69.11(18)
C(5)-C(1)-H(1)	126.0	C(8)-C(7)-H(7)	126.1
C(2)-C(1)-H(1)	126.0	C(6)-C(7)-H(7)	126.1
Co(1)-C(1)-H(1)	126.0	Co(1)-C(7)-H(7)	126.4
C(3)-C(2)-C(1)	108.1(4)	C(9)-C(8)-C(7)	108.2(3)
C(3)-C(2)-Co(1)	70.0(2)	C(9)-C(8)-Co(1)	69.49(18)
C(1)-C(2)-Co(1)	69.5(2)	C(7)-C(8)-Co(1)	69.29(19)
C(3)-C(2)-H(2)	125.9	C(9)-C(8)-H(8)	125.9
C(1)-C(2)-H(2)	125.9	C(7)-C(8)-H(8)	125.9
Co(1)-C(2)-H(2)	126.2	Co(1)-C(8)-H(8)	126.9
C(4)-C(3)-C(2)	107.8(3)	C(8)-C(9)-C(10)	108.3(3)
C(4)-C(3)-Co(1)	69.7(2)	C(8)-C(9)-Co(1)	69.81(19)
C(2)-C(3)-Co(1)	69.5(2)	C(10)-C(9)-Co(1)	69.81(18)
C(4)-C(3)-H(3)	126.1	C(8)-C(9)-H(9)	125.9
C(2)-C(3)-H(3)	126.1	C(10)-C(9)-H(9)	125.9
Co(1)-C(3)-H(3)	126.3	Co(1)-C(9)-H(9)	126.1
C(3)-C(4)-C(5)	108.2(3)	C(6)-C(10)-C(9)	107.4(3)

C(6)-C(10)-Au(1)	127.2(2)	C(9)-C(10)-Co(1)	69.24(18)
C(9)-C(10)-Au(1)	125.4(2)	Au(1)-C(10)-Co(1)	128.29(15)
C(6)-C(10)-Co(1)	68.73(18)		

Table 10. Anisotropic displacement parameters (Å^2 x 10^3) for **3**. The anisotropic displacement factor exponent takes the form: $-2\pi^2[h^2 a^{*2}U^{11} + ... + 2 h k a^* b^* U^{12}]$

	U^{11}	U^{22}	U^{33}	U^{23}	U^{13}	U^{12}
Au(1)	22(1)	28(1)	26(1)	-2(1)	-1(1)	-1(1)
Co(1)	22(1)	26(1)	25(1)	1(1)	-1(1)	0(1)
Cl(1)	31(1)	41(1)	38(1)	-7(1)	1(1)	-11(1)
Cl(2)	54(1)	51(1)	35(1)	-3(1)	-1(1)	-12(1)
Cl(3)	41(1)	56(1)	36(1)	-2(1)	6(1)	-11(1)
C(1)	35(2)	51(2)	43(2)	16(2)	10(2)	20(2)
C(2)	21(2)	65(3)	48(2)	-6(2)	-2(2)	4(2)
C(3)	33(2)	46(2)	55(3)	6(2)	19(2)	-2(2)
C(4)	38(2)	52(2)	31(2)	0(2)	8(2)	7(2)
C(5)	36(2)	32(2)	47(2)	-6(2)	12(2)	5(2)
C(6)	25(2)	37(2)	27(2)	-3(1)	4(1)	4(1)
C(7)	30(2)	39(2)	35(2)	-14(2)	-5(1)	2(2)
C(8)	30(2)	25(2)	48(2)	-3(1)	-4(2)	1(1)
C(9)	27(2)	27(2)	36(2)	2(1)	-4(1)	2(1)
C(10)	22(2)	26(2)	27(2)	-2(1)	0(1)	3(1)

Table 11. Hydrogen coordinates (x 10^4) and isotropic displacement parameters (Å^2 x 10^3) for **3**.

	x	y	z	U(eq)
H(1)	822	3926	4934	52
H(2)	-597	1807	5056	53
H(3)	-190	918	6832	53
H(4)	1468	2492	7809	49
H(5)	2105	4347	6632	46
H(6)	3877	2608	4247	36
H(7)	2419	488	4149	42
H(8)	2769	-671	5842	42
H(9)	4422	729	6989	36

Table 12. Torsion angles [°] for **3**.

C(5)-C(1)-C(2)-C(3)	0.0(4)	C(10)-C(6)-C(7)-Co(1)	59.9(2)
Co(1)-C(1)-C(2)-C(3)	59.5(3)	C(6)-C(7)-C(8)-C(9)	0.2(4)
C(5)-C(1)-C(2)-Co(1)	-59.6(3)	Co(1)-C(7)-C(8)-C(9)	-58.7(2)
C(1)-C(2)-C(3)-C(4)	0.2(4)	C(6)-C(7)-C(8)-Co(1)	58.9(2)
Co(1)-C(2)-C(3)-C(4)	59.4(3)	C(7)-C(8)-C(9)-C(10)	-0.8(4)
C(1)-C(2)-C(3)-Co(1)	-59.2(3)	Co(1)-C(8)-C(9)-C(10)	-59.4(2)
C(2)-C(3)-C(4)-C(5)	-0.3(4)	C(7)-C(8)-C(9)-Co(1)	58.6(2)
Co(1)-C(3)-C(4)-C(5)	59.0(3)	C(7)-C(6)-C(10)-C(9)	-1.0(4)
C(2)-C(3)-C(4)-Co(1)	-59.3(3)	Co(1)-C(6)-C(10)-C(9)	58.7(2)
C(2)-C(1)-C(5)-C(4)	-0.2(4)	C(7)-C(6)-C(10)-Au(1)	177.7(2)
Co(1)-C(1)-C(5)-C(4)	-59.7(3)	Co(1)-C(6)-C(10)-Au(1)	-122.6(2)
C(2)-C(1)-C(5)-Co(1)	59.6(3)	C(7)-C(6)-C(10)-Co(1)	-59.6(2)
C(3)-C(4)-C(5)-C(1)	0.3(4)	C(8)-C(9)-C(10)-C(6)	1.1(4)
Co(1)-C(4)-C(5)-C(1)	59.6(2)	Co(1)-C(9)-C(10)-C(6)	-58.3(2)
C(3)-C(4)-C(5)-Co(1)	-59.3(3)	C(8)-C(9)-C(10)-Au(1)	-177.6(2)
C(10)-C(6)-C(7)-C(8)	0.5(4)	Co(1)-C(9)-C(10)-Au(1)	122.9(2)
Co(1)-C(6)-C(7)-C(8)	-59.4(2)	C(8)-C(9)-C(10)-Co(1)	59.4(2)

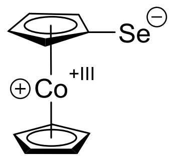

[267,07]
$C_{10}H_9CoSe$

Cobaltocenium selenolate (**5a**): All transformations were conducted using Schlenk techniques under argon atmosphere. 0.039 g of freshly prepared Na_2Se (0.305 mmol, 1.5 equiv.)[4] was suspended in 15 ml of dry THF. 0.090 g iodocobaltocenium iodide[1] (0.204 mmol, 1 equiv.) was added and the mixture was stirred at ambient temperature overnight. The colour changed from brown to intense violet. For purification via Schlenk filtration the reaction mixture was directly poured on a short column of neutral aluminium oxide and thoroughly washed with dry THF. *Note 6:* **5a** adsorbs red on Al_2O_3. Intense violet **5a** was eluted with several portions of dry CH_3CN until the eluate was nearly colourless. CH_3CN was evaporated *in vacuo* and **5a** was obtained in nearly quantitative yield. *Note 7:* Traces of unsubstituted cobaltocenium salt (~3% estimated by ^{1}H-NMR) and dicobaltoceniumyl diselenide (far below 1%; due to partial oxidation of **5a**) were found as impurities. ^{1}H-NMR (300 MHz, CD_3CN): δ 5.07 (s, 5H, Cp), 5.36 (broad-s, 4H, C2/C5 and C3/C4 of substituted Cp) ppm. ^{13}C-NMR (75 MHz, CD_3CN): δ 82.2 (C2/C5 of substituted Cp), 86.1 (Cp), 86.2 (C3/C4 of substituted Cp), 129.4 (*ipso*-carbon) ppm. ^{77}Se-NMR (57 MHz, CD_3CN): δ 258 (vs. Me_2Se), -1042 (vs. saturated H_2SeO_3 in D_2O) ppm. *Note 8:* The sample was measured for 1.3 h, 1,600 scans. IR (ATR): 3120 (ν_{C-H}), 2956, 2919, 2854, 1627, 1407 ($\nu_{C=C}$), 1260, 1133, 1103, 1020, 828 (ν_{C-Se}), 602, 579, 554, 520, 493, 453 ($\nu_{as(Co-ring)}$) cm^{-1}. **5a** was crystallized out of dry acetone. MS (ESI pos [m/z]): 267.92 $[M]^+$.

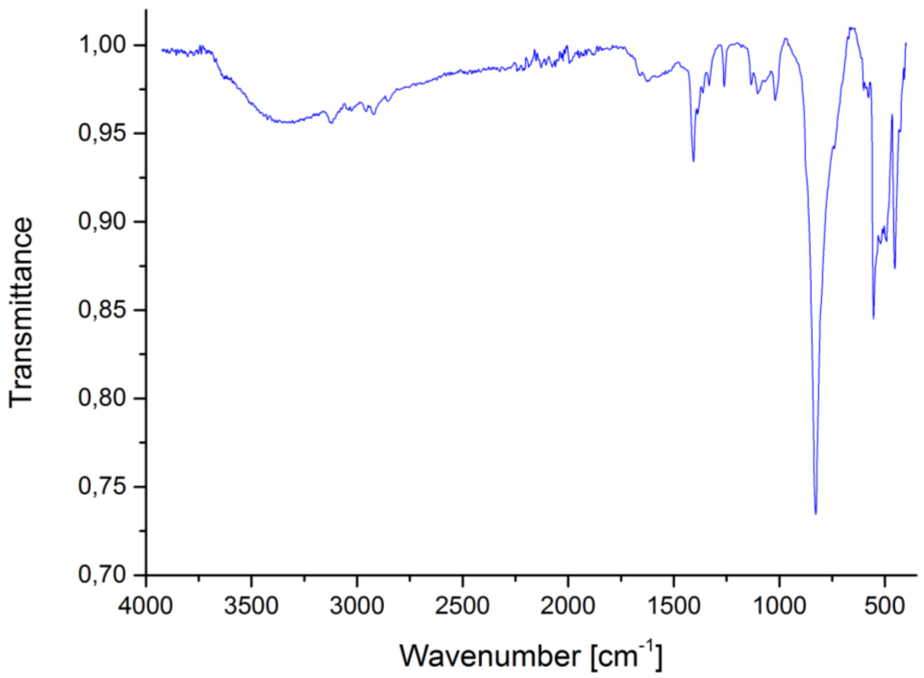

Figure S9. IR-spectrum (ATR, [cm^{-1}]) of **5a**.

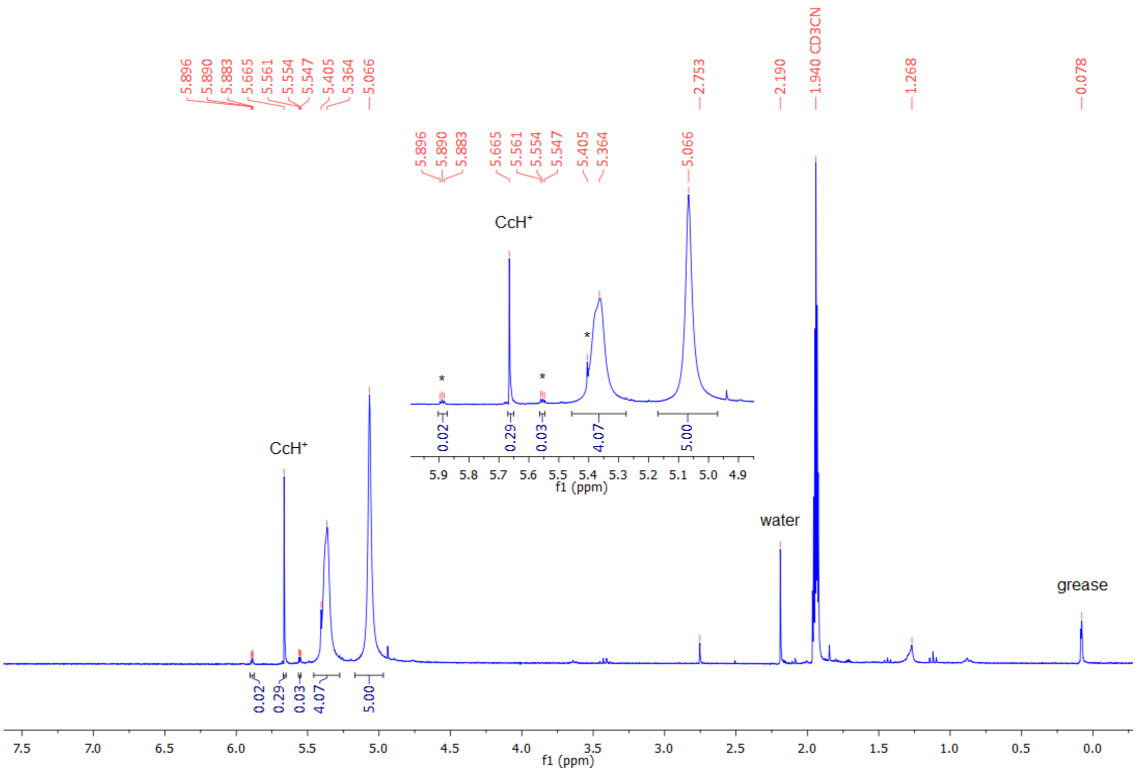

Figure S10. ^{1}H-NMR (300 MHz, CD_3CN, [ppm]) of **5a** (impurity of oxidation product marked).

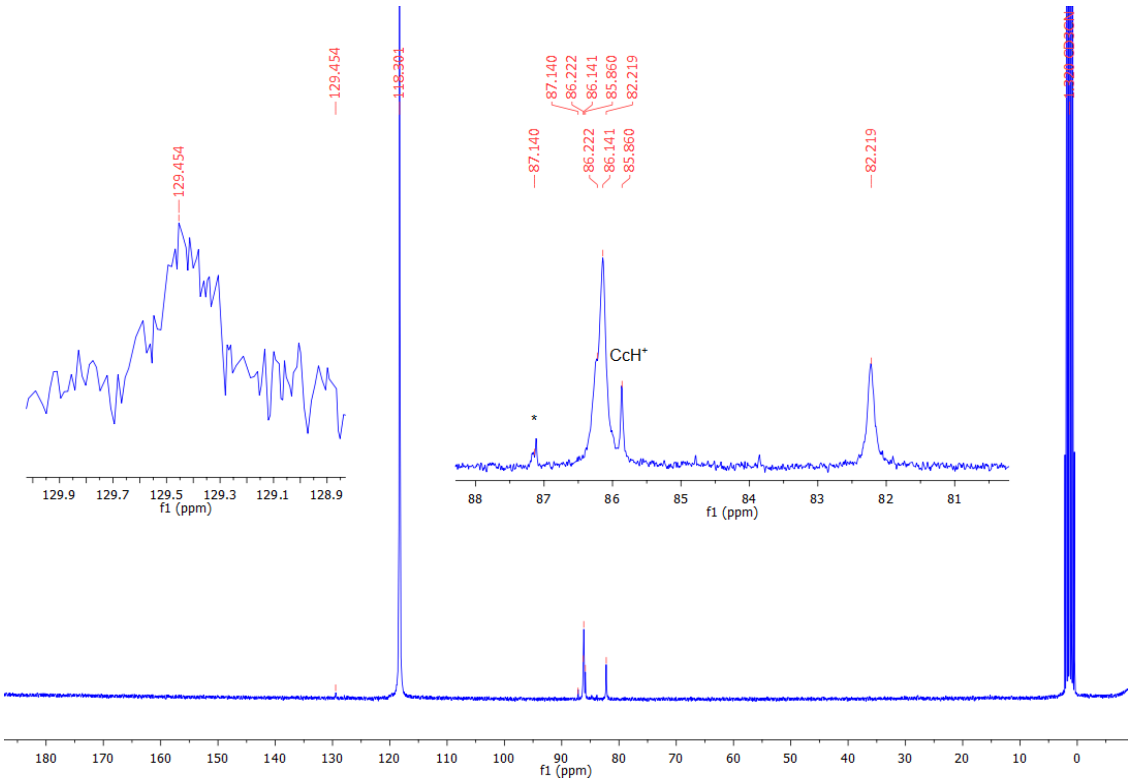

Figure S11. ^{13}C-NMR (75 MHz, CD_3CN, [ppm]) of **5a** (impurity of oxidation product marked).

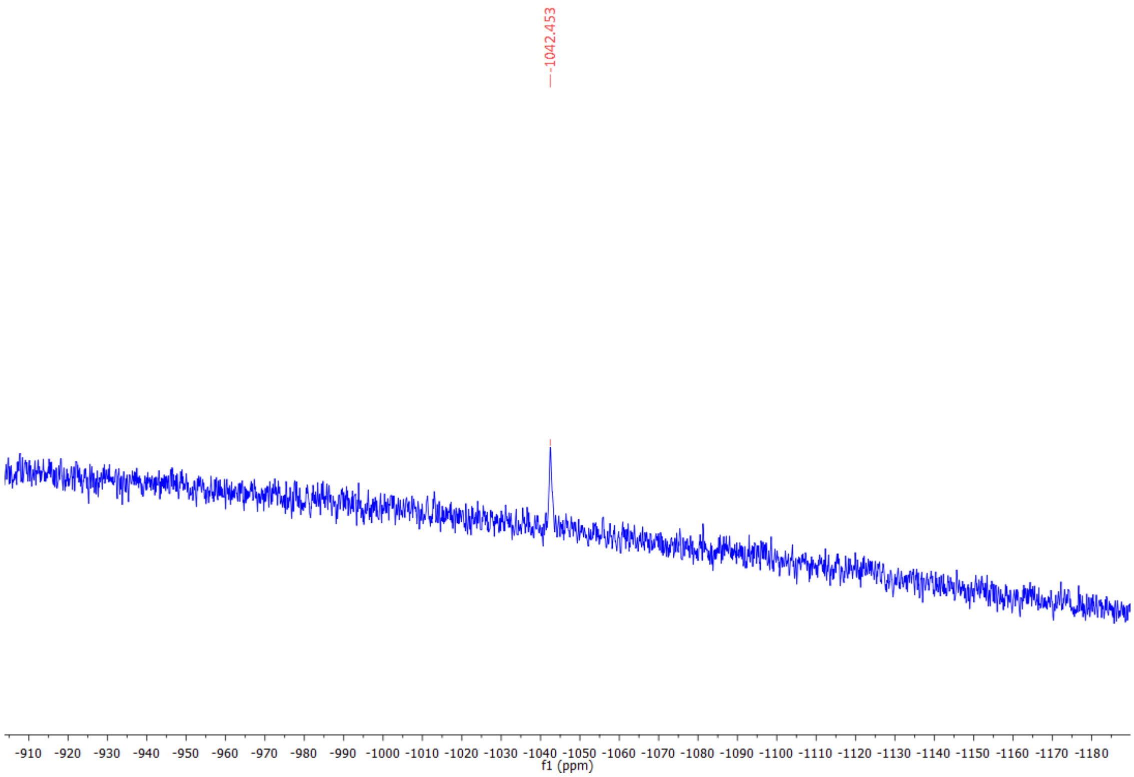

Figure S12. ^{77}Se-NMR (57 MHz, CD_3CN, [ppm]) of **5a** (vs. saturated H_2SeO_3 in D_2O).

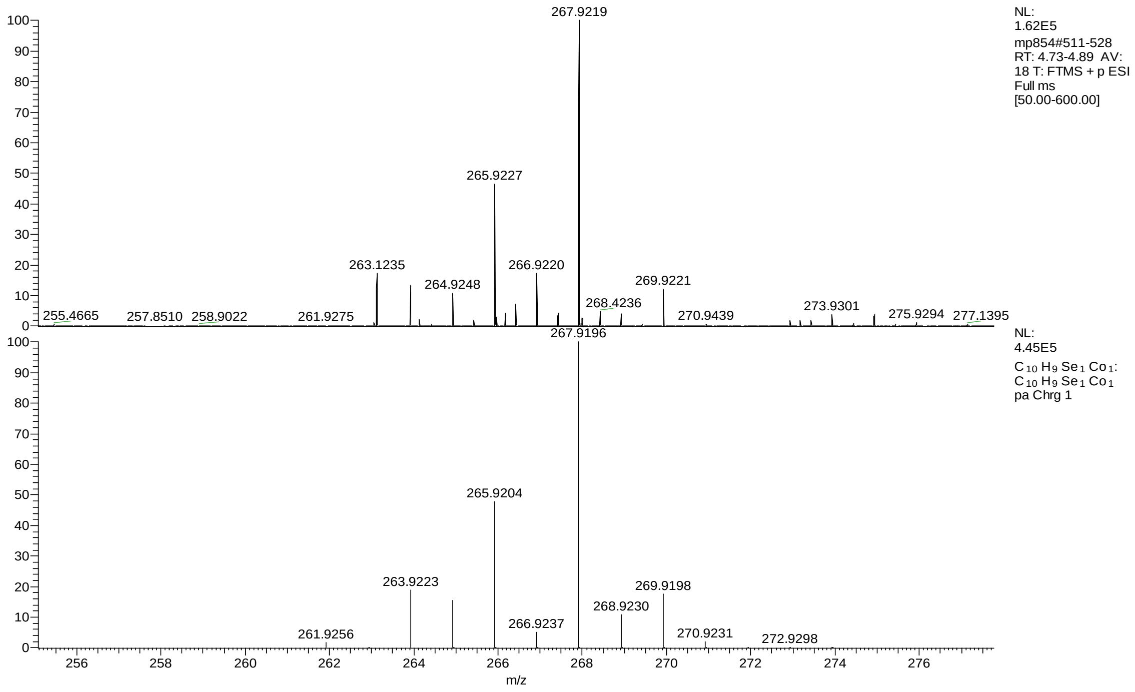

Figure S13. MS (ESI pos, [m/z]; *bottom*: simulated, *top*: experimental) of **5a**; accompanied by doubly charged fragments of the oxidation product.

Crystallographic data

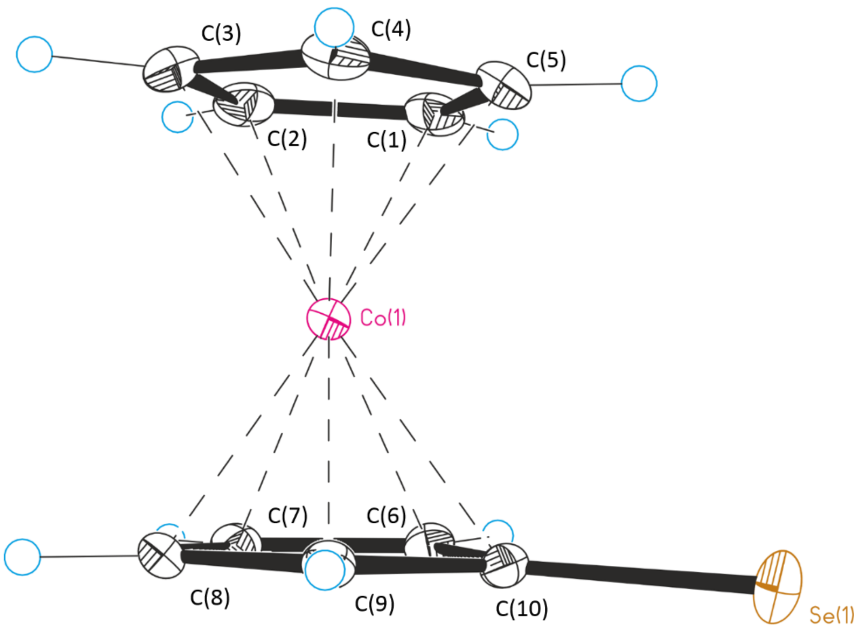

Figure S14. X-ray single crystal structure analysis of **5a**.

Table 13. Crystal data and structure refinement for **5a**.

Identification code	bi119	
Empirical formula	$C_{10}H_9CoSe$	
Formula weight	267.06	
Temperature	183(2) K	
Wavelength	0.71073 Å	
Crystal system	Monoclinic	
Space group	$P2_1/n$ (no. 14)	
Unit cell dimensions	*a* = 7.4182(12) Å	*α* = 90°
	b = 12.2609(12) Å	*ß* = 95.512(7)°
	c = 9.9706(16) Å	*γ* = 90°
Volume	902.7(2) $Å^3$	
Z	4	
Density (calculated)	1.965 Mg/m^3	
Absorption coefficient	5.867 mm^{-1}	
F(000)	520	

Crystal size	0.140 x 0.070 x 0.040 mm^3
Theta range for data collection	2.640 to 25.705°.
Index ranges	$-9<h<9$, $-14<k<14$, $-12<l<12$
Reflections collected	16750
Independent reflections	1717 [*R*(int) = 0.0285]
Completeness to theta = 25.242°	100.0 %
Absorption correction	Semi-empirical from equivalents
Max. and min. transmission	0.875 and 0.762
Refinement method	Full-matrix least-squares on F^2
Data / restraints / parameters	1717 / 0 / 109
Goodness-of-fit on F^2	1.084
Final R indices [I>2sigma(I)]	*R*1 = 0.0172, w*R*2 = 0.0410
R indices (all data)	*R*1 = 0.0202, w*R*2 = 0.0423
Extinction coefficient	n/a
Largest diff. peak and hole	0.253 and -0.423 e.Å^{-3}

Table 14. Atomic coordinates (x 10^4) and equivalent isotropic displacement parameters (Å^2 x 10^3) for **5a**. U(eq) is defined as one third of the trace of the orthogonalized U^{ij} tensor.

	x	y	z	U(eq)
Se(1)	8605(1)	7004(1)	5538(1)	28(1)
Co(1)	7157(1)	5714(1)	2364(1)	16(1)
C(1)	9533(3)	5164(2)	1762(2)	27(1)
C(2)	8074(3)	4869(2)	796(2)	28(1)
C(3)	7207(3)	5838(2)	317(2)	28(1)
C(4)	8119(3)	6733(2)	980(2)	28(1)
C(5)	9564(3)	6316(2)	1868(2)	28(1)
C(6)	7007(3)	5022(2)	4200(2)	21(1)
C(7)	5494(3)	4721(2)	3284(2)	24(1)
C(8)	4577(2)	5692(2)	2848(2)	25(1)
C(9)	5510(2)	6586(2)	3497(2)	22(1)
C(10)	7003(2)	6182(2)	4395(2)	19(1)

Table 15. Bond lengths [Å] and angles [°] for **5a**.

Se(1)-C(10)	1.8613(19)	C(7)-Co(1)-C(8)	41.16(8)
Co(1)-C(7)	2.0161(18)	C(7)-Co(1)-C(6)	41.27(8)
Co(1)-C(8)	2.0189(19)	C(8)-Co(1)-C(6)	69.10(8)
Co(1)-C(6)	2.0305(18)	C(7)-Co(1)-C(1)	121.34(8)
Co(1)-C(1)	2.0311(19)	C(8)-Co(1)-C(1)	159.36(8)
Co(1)-C(5)	2.0359(19)	C(6)-Co(1)-C(1)	104.58(8)
Co(1)-C(4)	2.040(2)	C(7)-Co(1)-C(5)	156.74(8)
Co(1)-C(9)	2.0439(18)	C(8)-Co(1)-C(5)	159.50(8)
Co(1)-C(2)	2.0455(19)	C(6)-Co(1)-C(5)	119.73(8)
Co(1)-C(3)	2.051(2)	C(1)-Co(1)-C(5)	40.76(8)
Co(1)-C(10)	2.1183(18)	C(7)-Co(1)-C(4)	161.19(8)
C(1)-C(5)	1.416(3)	C(8)-Co(1)-C(4)	124.85(8)
C(1)-C(2)	1.425(3)	C(6)-Co(1)-C(4)	156.73(8)
C(1)-H(1)	0.9500	C(1)-Co(1)-C(4)	68.52(8)
C(2)-C(3)	1.412(3)	C(5)-Co(1)-C(4)	40.71(8)
C(2)-H(2)	0.9500	C(7)-Co(1)-C(9)	68.90(8)
C(3)-C(4)	1.419(3)	C(8)-Co(1)-C(9)	40.89(8)
C(3)-H(3)	0.9500	C(6)-Co(1)-C(9)	68.45(8)
C(4)-C(5)	1.418(3)	C(1)-Co(1)-C(9)	156.76(8)
C(4)-H(4)	0.9500	C(5)-Co(1)-C(9)	121.97(8)
C(5)-H(5)	0.9500	C(4)-Co(1)-C(9)	108.67(8)
C(6)-C(7)	1.426(3)	C(7)-Co(1)-C(2)	107.74(8)
C(6)-C(10)	1.436(3)	C(8)-Co(1)-C(2)	124.64(8)
C(6)-H(6)	0.9500	C(6)-Co(1)-C(2)	121.91(8)
C(7)-C(8)	1.418(3)	C(1)-Co(1)-C(2)	40.92(8)
C(7)-H(7)	0.9500	C(5)-Co(1)-C(2)	68.54(8)
C(8)-C(9)	1.419(3)	C(4)-Co(1)-C(2)	68.26(9)
C(8)-H(8)	0.9500	C(9)-Co(1)-C(2)	161.37(8)
C(9)-C(10)	1.443(3)	C(7)-Co(1)-C(3)	124.60(8)
C(9)-H(9)	0.9500	C(8)-Co(1)-C(3)	110.31(8)
		C(6)-Co(1)-C(3)	159.41(8)
		C(1)-Co(1)-C(3)	68.38(8)
		C(5)-Co(1)-C(3)	68.37(8)
		C(4)-Co(1)-C(3)	40.59(8)

C(9)-Co(1)-C(3)	125.47(8)	C(1)-C(5)-Co(1)	69.43(11)
C(2)-Co(1)-C(3)	40.32(8)	C(4)-C(5)-Co(1)	69.81(11)
C(7)-Co(1)-C(10)	68.73(8)	C(1)-C(5)-H(5)	126.0
C(8)-Co(1)-C(10)	68.62(7)	C(4)-C(5)-H(5)	126.0
C(6)-Co(1)-C(10)	40.43(7)	Co(1)-C(5)-H(5)	126.3
C(1)-Co(1)-C(10)	120.03(8)	C(7)-C(6)-C(10)	109.35(17)
C(5)-Co(1)-C(10)	105.26(8)	C(7)-C(6)-Co(1)	68.82(11)
C(4)-Co(1)-C(10)	122.35(8)	C(10)-C(6)-Co(1)	73.08(10)
C(9)-Co(1)-C(10)	40.52(7)	C(7)-C(6)-H(6)	125.3
C(2)-Co(1)-C(10)	156.98(8)	C(10)-C(6)-H(6)	125.3
C(3)-Co(1)-C(10)	159.91(8)	Co(1)-C(6)-H(6)	124.3
C(5)-C(1)-C(2)	107.96(18)	C(8)-C(7)-C(6)	107.67(17)
C(5)-C(1)-Co(1)	69.80(11)	C(8)-C(7)-Co(1)	69.53(11)
C(2)-C(1)-Co(1)	70.08(11)	C(6)-C(7)-Co(1)	69.90(10)
C(5)-C(1)-H(1)	126.0	C(8)-C(7)-H(7)	126.2
C(2)-C(1)-H(1)	126.0	C(6)-C(7)-H(7)	126.2
Co(1)-C(1)-H(1)	125.7	Co(1)-C(7)-H(7)	126.0
C(3)-C(2)-C(1)	107.91(18)	C(7)-C(8)-C(9)	108.08(17)
C(3)-C(2)-Co(1)	70.04(11)	C(7)-C(8)-Co(1)	69.31(11)
C(1)-C(2)-Co(1)	69.00(11)	C(9)-C(8)-Co(1)	70.50(10)
C(3)-C(2)-H(2)	126.0	C(7)-C(8)-H(8)	126.0
C(1)-C(2)-H(2)	126.0	C(9)-C(8)-H(8)	126.0
Co(1)-C(2)-H(2)	126.5	Co(1)-C(8)-H(8)	125.8
C(2)-C(3)-C(4)	108.14(19)	C(8)-C(9)-C(10)	109.23(17)
C(2)-C(3)-Co(1)	69.63(11)	C(8)-C(9)-Co(1)	68.61(11)
C(4)-C(3)-Co(1)	69.30(11)	C(10)-C(9)-Co(1)	72.51(10)
C(2)-C(3)-H(3)	125.9	C(8)-C(9)-H(9)	125.4
C(4)-C(3)-H(3)	125.9	C(10)-C(9)-H(9)	125.4
Co(1)-C(3)-H(3)	126.7	Co(1)-C(9)-H(9)	125.1
C(5)-C(4)-C(3)	108.06(18)	C(6)-C(10)-C(9)	105.52(16)
C(5)-C(4)-Co(1)	69.48(11)	C(6)-C(10)-Se(1)	127.54(14)
C(3)-C(4)-Co(1)	70.11(11)	C(9)-C(10)-Se(1)	126.93(14)
C(5)-C(4)-H(4)	126.0	C(6)-C(10)-Co(1)	66.50(10)
C(3)-C(4)-H(4)	126.0	C(9)-C(10)-Co(1)	66.97(10)
Co(1)-C(4)-H(4)	126.0	Se(1)-C(10)-Co(1)	129.94(9)
C(1)-C(5)-C(4)	107.92(18)		

Table 16. Anisotropic displacement parameters (Å^2 x 10^3) for **5a**. The anisotropic displacement factor exponent takes the form: $-2\pi^2$[h^2 $a^{*2}U^{11}$ + ... + 2 h k a* b* U^{12}]

	U^{11}	U^{22}	U^{33}	U^{23}	U^{13}	U^{12}
Se(1)	30(1)	24(1)	26(1)	-5(1)	-9(1)	1(1)
Co(1)	15(1)	19(1)	16(1)	0(1)	2(1)	-1(1)
C(1)	20(1)	33(1)	30(1)	4(1)	11(1)	6(1)
C(2)	32(1)	28(1)	26(1)	-6(1)	14(1)	0(1)
C(3)	28(1)	39(1)	16(1)	1(1)	5(1)	-1(1)
C(4)	28(1)	26(1)	30(1)	7(1)	11(1)	-1(1)
C(5)	19(1)	36(1)	29(1)	0(1)	8(1)	-7(1)
C(6)	24(1)	21(1)	18(1)	4(1)	3(1)	0(1)
C(7)	26(1)	26(1)	22(1)	-1(1)	7(1)	-10(1)
C(8)	15(1)	39(1)	21(1)	-2(1)	3(1)	-2(1)
C(9)	18(1)	28(1)	20(1)	0(1)	4(1)	5(1)
C(10)	19(1)	24(1)	16(1)	0(1)	3(1)	1(1)

Table 17. Hydrogen coordinates (x 10^4) and isotropic displacement parameters (Å^2 x 10^3) for **5a**.

	x	y	z	U(eq)
H(1)	10343	4674	2250	33
H(2)	7743	4149	522	33
H(3)	6189	5883	-337	33
H(4)	7816	7481	852	33
H(5)	10403	6735	2433	34
H(6)	7881	4532	4616	25
H(7)	5160	4000	3014	29
H(8)	3522	5736	2230	29
H(9)	5201	7332	3362	26

Table 18. Torsion angles [°] for **5a**.

C(5)-C(1)-C(2)-C(3)	-0.3(2)	C(10)-C(6)-C(7)-Co(1)	62.16(13)
Co(1)-C(1)-C(2)-C(3)	59.45(13)	C(6)-C(7)-C(8)-C(9)	-0.3(2)
C(5)-C(1)-C(2)-Co(1)	-59.77(13)	Co(1)-C(7)-C(8)-C(9)	-60.09(13)
C(1)-C(2)-C(3)-C(4)	0.0(2)	C(6)-C(7)-C(8)-Co(1)	59.74(13)
Co(1)-C(2)-C(3)-C(4)	58.78(13)	C(7)-C(8)-C(9)-C(10)	-2.1(2)
C(1)-C(2)-C(3)-Co(1)	-58.79(13)	Co(1)-C(8)-C(9)-C(10)	-61.42(13)
C(2)-C(3)-C(4)-C(5)	0.3(2)	C(7)-C(8)-C(9)-Co(1)	59.35(13)
Co(1)-C(3)-C(4)-C(5)	59.32(14)	C(7)-C(6)-C(10)-C(9)	-3.8(2)
C(2)-C(3)-C(4)-Co(1)	-58.99(13)	Co(1)-C(6)-C(10)-C(9)	55.70(12)
C(2)-C(1)-C(5)-C(4)	0.5(2)	C(7)-C(6)-C(10)-Se(1)	177.20(13)
Co(1)-C(1)-C(5)-C(4)	-59.42(14)	Co(1)-C(6)-C(10)-Se(1)	-123.27(15)
C(2)-C(1)-C(5)-Co(1)	59.95(13)	C(7)-C(6)-C(10)-Co(1)	-59.53(13)
C(3)-C(4)-C(5)-C(1)	-0.5(2)	C(8)-C(9)-C(10)-C(6)	3.6(2)
Co(1)-C(4)-C(5)-C(1)	59.18(13)	Co(1)-C(9)-C(10)-C(6)	-55.40(12)
C(3)-C(4)-C(5)-Co(1)	-59.72(14)	C(8)-C(9)-C(10)-Se(1)	-177.41(13)
C(10)-C(6)-C(7)-C(8)	2.7(2)	Co(1)-C(9)-C(10)-Se(1)	123.58(14)
Co(1)-C(6)-C(7)-C(8)	-59.51(13)	C(8)-C(9)-C(10)-Co(1)	59.02(13)

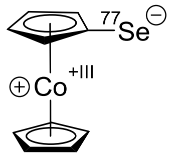

[265,03]
$C_{10}H_9Co^{77}Se$

^{77}Se-cobaltocenium selenolate (**5b**): The same procedure as for **5a** was applied, however, $Na_2{}^{77}Se$ was synthesized from 0.010 g of 99.5% enriched ^{77}Se (0.130 mmol, 1,1 equiv.) and 0.012 g of sodium (0.520 mmol, 4 equiv.), accompanied by 10 mol% of naphthalene in dry 1,4-dioxane. _Note 9:_ Highly stable isotopically pure ^{77}Se was freshly ground and required an excess of sodium, concomitant with a more cumbersome synthesis (72h ultrasound and 24h reflux in dry 1,4-dioxane), compared to its naturally occurring analogue,[4] obtained as fine powder. The excessive amount of sodium was consumed most likely as a result of selenium dioxide, formed during the grinding process. **5b** was obtained in nearly quantitative yield. _Note 10:_ Unsubstituted cobaltocenium salt (~10% estimated by ^{1}H-NMR) and dicobaltoceniumyl diselenide (far below 1%; due to partial oxidation of **5a**) occurred as impurities. The NMR solvent was afore purified and degassed. ^{1}H-NMR (300 MHz, CD_3CN): δ 5.06 (s, 5H, Cp), 5.37 (broad-s, 4H, C2/C5 and C3/C4 of substituted Cp) ppm. ^{13}C-NMR (75 MHz, CD_3CN): δ 82.2 (C2/C5 of substituted Cp), 86.2 (broad, Cp and C3/C4 of substituted Cp), 129.3 (d, $^1J_{C\text{-}Se}$ = 199 Hz, *ipso*-carbon) ppm. _Note 11:_ The measurement of the *ispo*-carbon duplet required 68 h, 76.500 scans and addition of $Cr(acac)_3$. ^{77}Se-NMR (57 MHz, CD_3CN): δ 256 (vs. Me_2Se), -1044 (vs. saturated H_2SeO_3 in D_2O) ppm. _Note 12:_ Due to isotopic pure ^{77}Se, the measurement of the ^{77}Se-NMR was more sensitive and could be conducted in 1.5 h, 2,752 scans. IR (ATR): 3124 ($\nu_{C\text{–}H}$), 2962, 1408 ($\nu_{C=C}$), 1261, 1095, 1019, 828 ($\nu_{C\text{–}Se}$), 554, 516, 493, 452 ($\nu_{as(Co\text{-}ring)}$) cm^{-1}. MS (ESI pos [m/z]): 264.92 [M]$^+$.

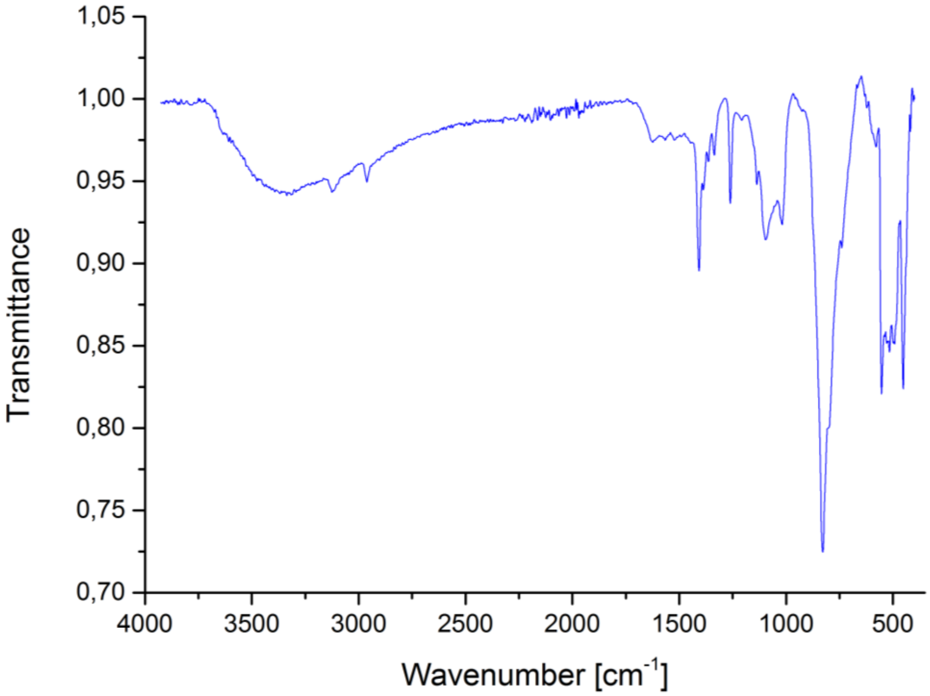

Figure S15. IR-spectrum (ATR, [cm^{-1}]) of **5b**.

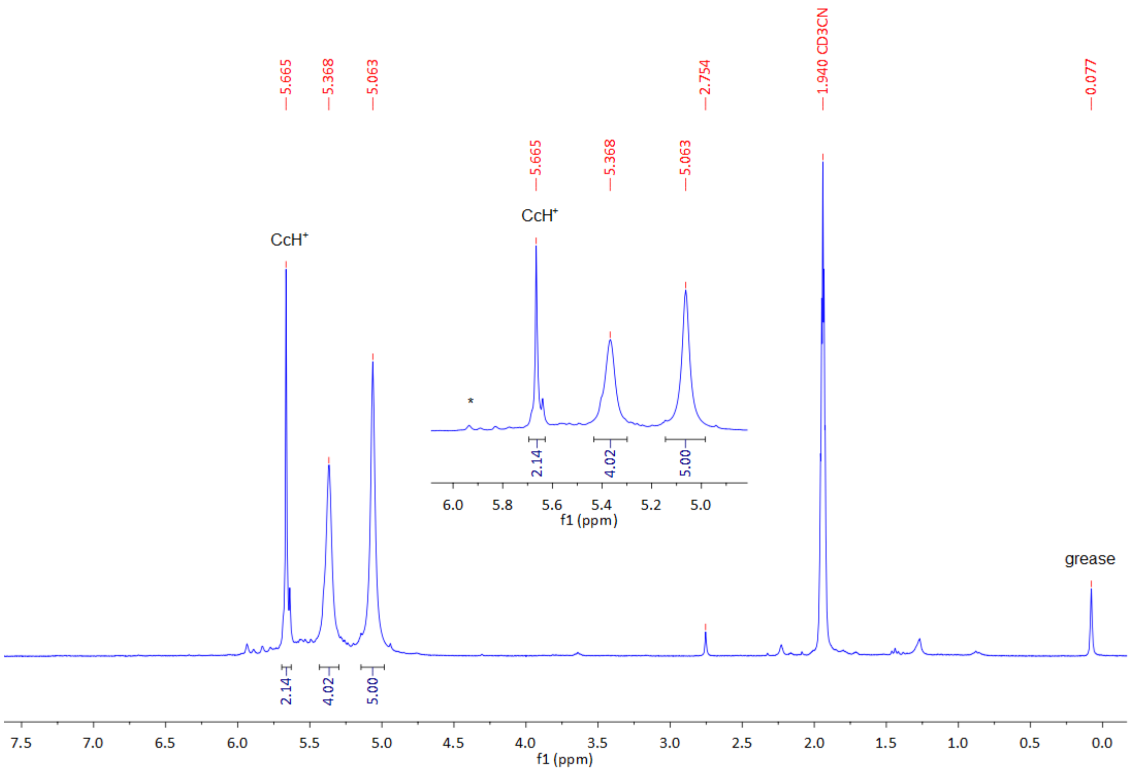

Figure S16. ^{1}H-NMR (300 MHz, CD_3CN, [ppm]) of **5b** (impurity of oxidation product marked).

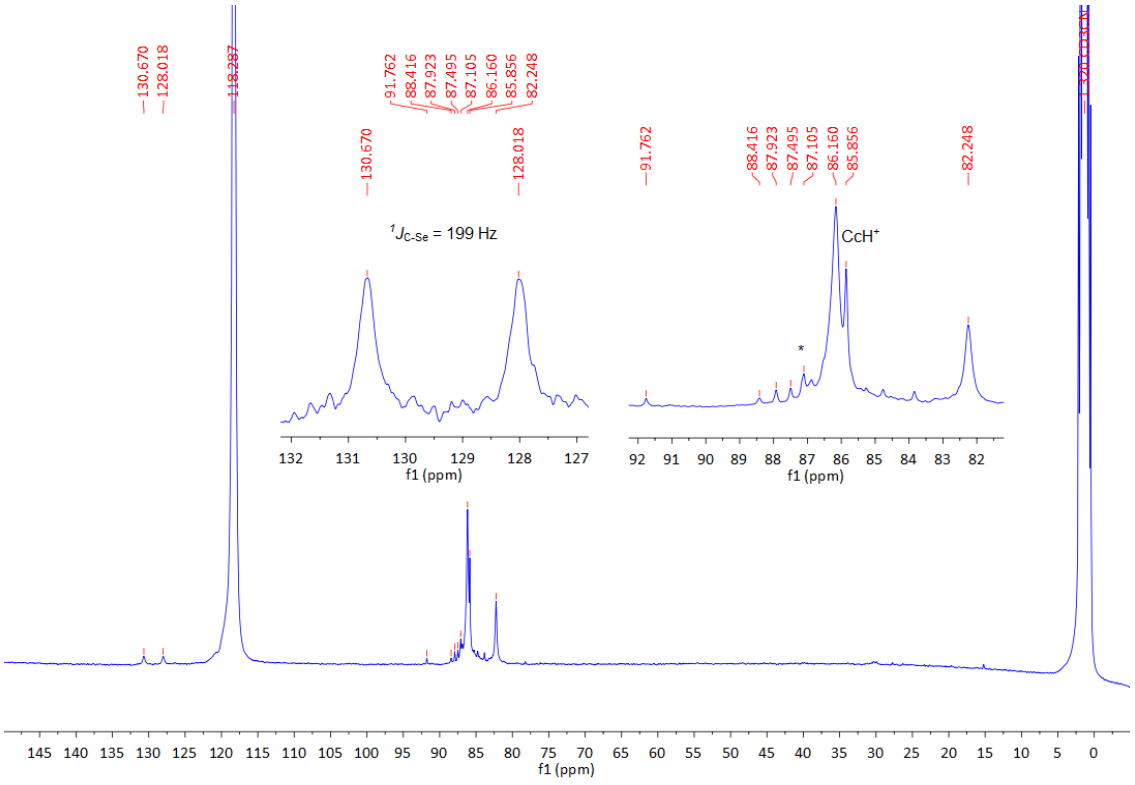

Figure S17. ^{13}C-NMR (75 MHz, CD_3CN, [ppm]) of **5b** (impurity of oxidation product marked).

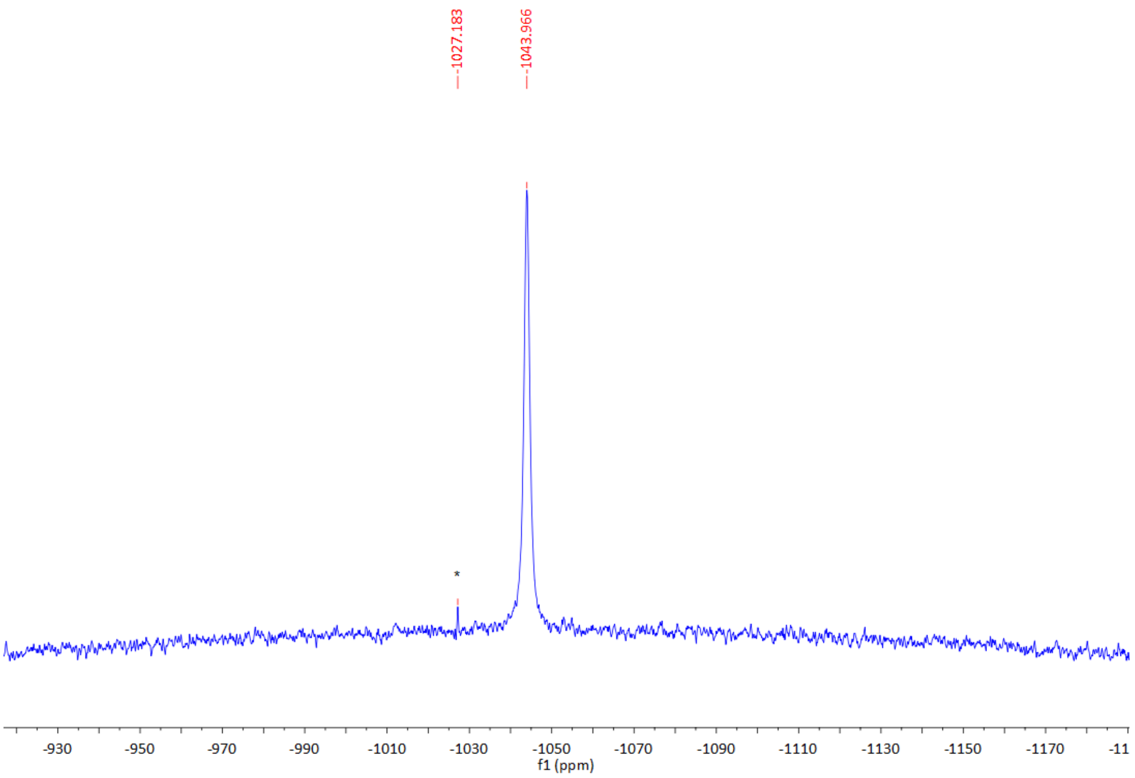

Figure S18. ^{77}Se-NMR (57 MHz, CD_3CN, [ppm]) of **5b** vs. saturated H_2SeO_3 in D_2O (impurity of oxidation product marked).

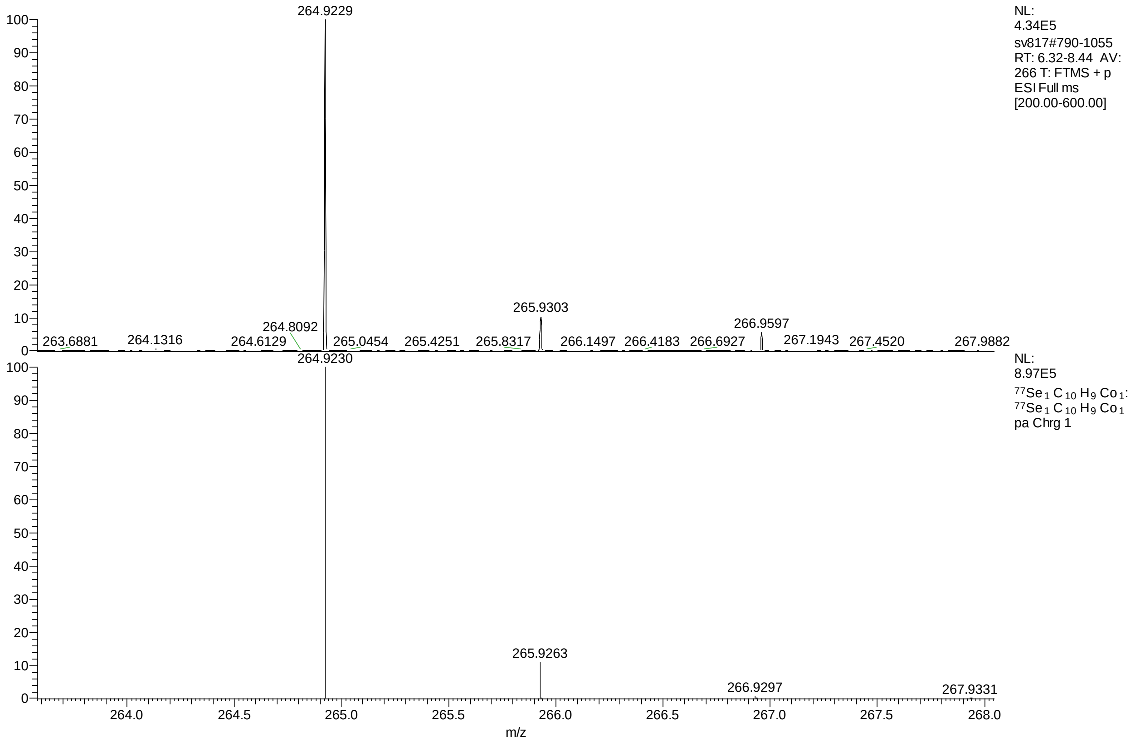

Figure S19. MS (ESI pos, [m/z]; *bottom*: simulated, *top*: experimental) of **5b**.

Electrochemical Section

Electrochemical measurements. Electrochemical experiments were executed in a home-built cylindrical vacuum-tight one-compartment cell. A spiral-shaped Pt wire and a Ag wire as the counter and pseudoreference electrodes are sealed into glass capillaries and fixed by Quickfit screws via standard joints. A platinum electrode is introduced as the working electrode through the top port via a Teflon screw cap with a suitable fitting. It is polished with first 1 µm and then 0.25 µm diamond paste before measurements. The cell can be attached to a conventional Schlenk line via a side arm equipped with a Teflon screw valve, allowing experiments to be performed under an argon atmosphere with approximately 5 mL of analyte solution. A 0.1 M solution of NBu_4^+ PF_6^- in THF was used as the supporting electrolyte. Referencing was done with addition of an appropriate amount of ferrocene (Cp_2Fe) as an internal standard to the analyte solution after all data of interest had been acquired. Representative sets of scans were repeated with the added standard. Electrochemical data were acquired with a computer controlled BASi CV50 potentiostat.

Spectroelectrochemistry (in THF, NBu_4^+ PF_6^-, 0.1 M at r. t.) was performed with an optically transparent thin-layer electrochemical (OTTLE) cell was home-built and followed the design of Hartl et al.[5] It comprised a Pt working and counter electrode and a thin silver wire as a pseudoreference electrode sandwiched between two CaF_2 windows of a conventional liquid IR cell. The working electrode is positioned in the center of the spectrometer beam.

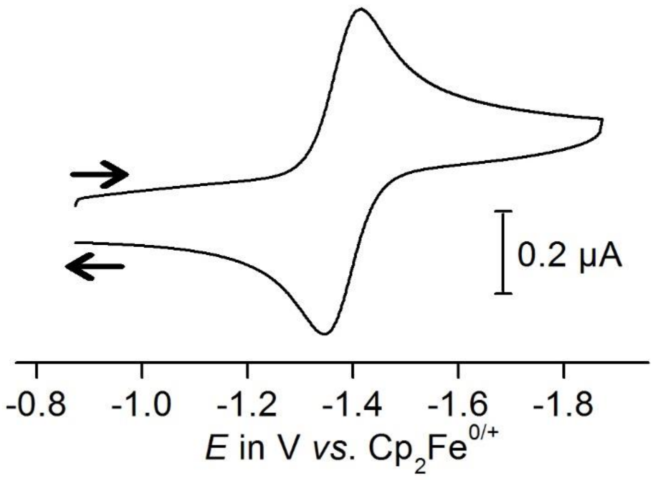

Figure S20. Cyclic voltammogram showing the first, reversible one-electron reduction wave of complex **2** (THF/NBu_4^+ PF_6^- 0.1 M, v = 0.1 V/s) at r. t).

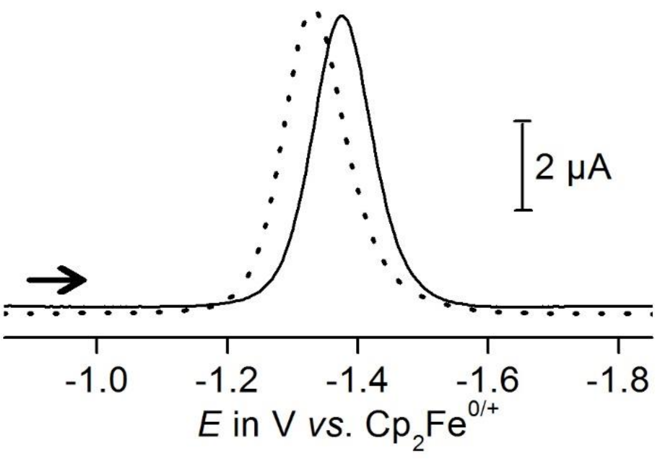

Figure S21. Comparison of square-wave voltammograms of compound **2** (solid line) and of cobaltocenium hexafluorophosphate (dotted line) in THF/NBu_4^+ PF_6^- 0.1 M at r. t.

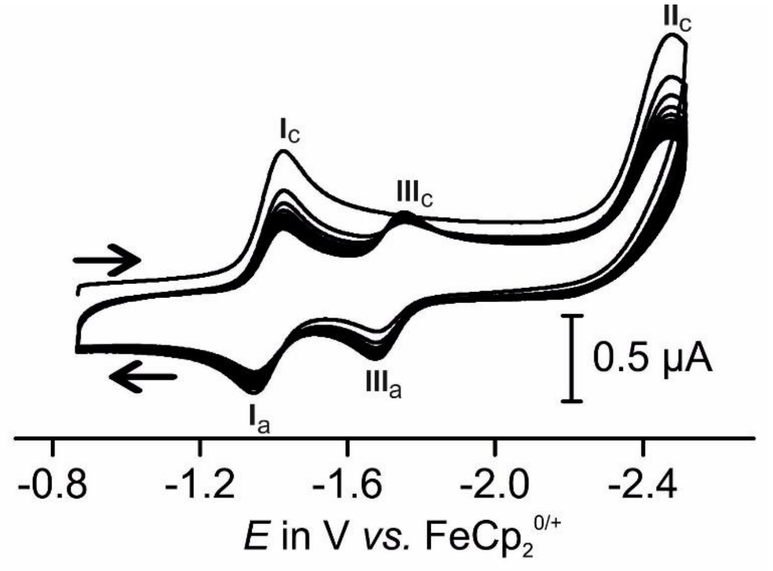

Figure S22. Cyclic voltammogram of complex **2** showing seven full cycles recorded after the initial cycle (THF/NBu_4^+ PF_6^- 0.1 M, *v* = 0.2 V/s) at r. t) with formation of the new reversible couple III_c/III_a after traversing peak II_c.

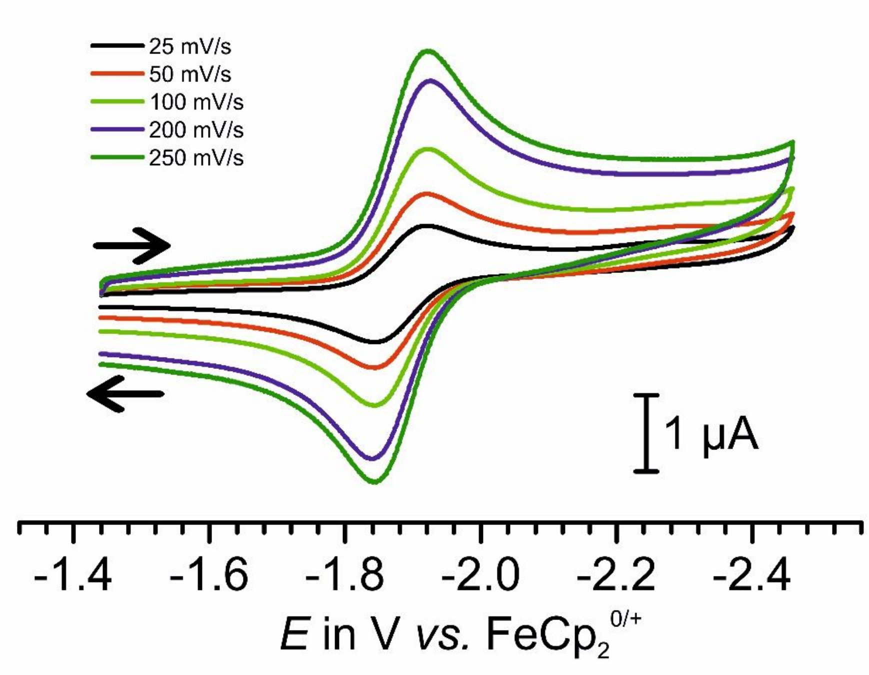

Figure S23. Cyclic voltammograms showing the reversible one-electron reduction wave of complex **5a** (THF/NBu_4^+ PF_6^- 0.1 M, r. t) at various sweep rates.

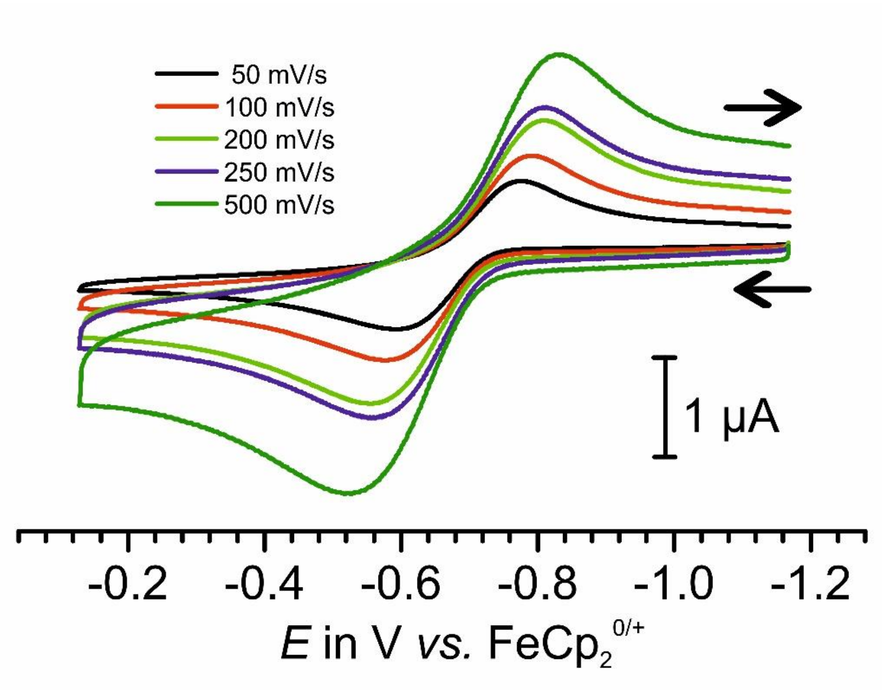

Figure S24. Cyclic voltammograms showing the quasireversible one-electron oxidation wave of complex **5a** (THF/NBu_4^+ PF_6^- 0.1 M, r. t) at various sweep rates.

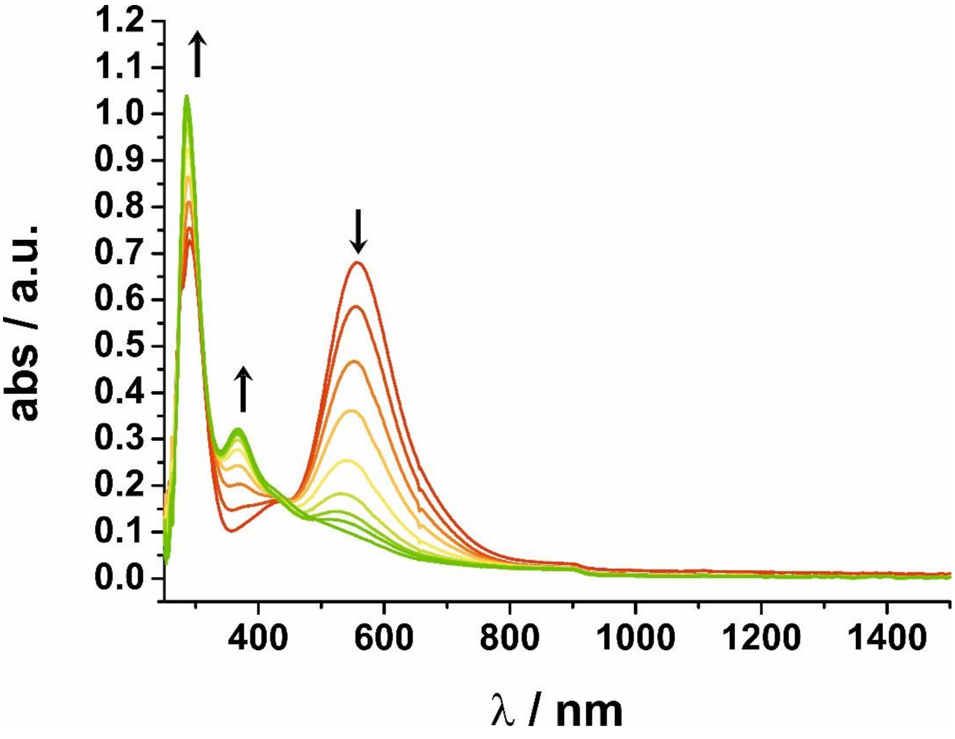

Figure S25. Spectroscopic changes on one-electron oxidation of complex **5a** in THF/NBu_4^+ PF_6^- 0.1 M, at r. t. inside a thin-layer electrochemical cell.

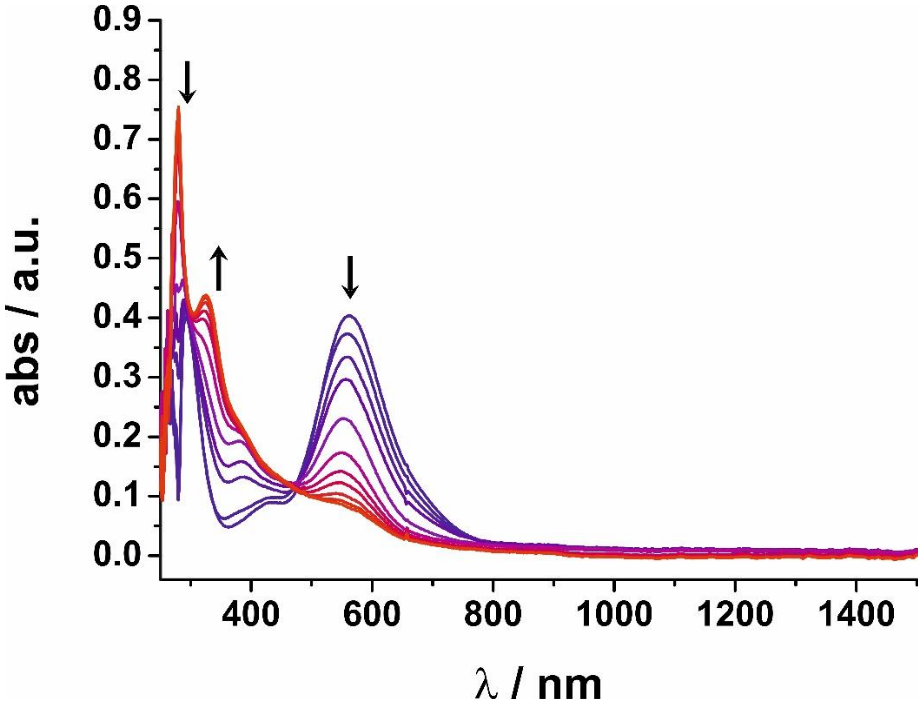

Figure S26. Spectroscopic changes on one-electron reduction of complex **5a** in THF/NBu_4^+ PF_6^- 0.1 M, at r. t. inside a thin-layer electrochemical cell.

Computational Section

Computational methodology. Structures of the free CcC ligand were derived from the X-ray crystal structure of $(CcC)Au(CN)_3$ and subjected to structure optimization. Density functional theory calculations were performed with the program suite Turbomole.[6] Two different density functionals, the pure BP86[7,8] as well as the hybrid PBE0[9] functional with 25% Hartree-Fock exchange were used in combination with the Schäfer et al. triple-zeta basis set.[10] For Au, an effective core potential was provided for the inner electrons.[11] A hybrid and a non-hybrid density functional were chosen because it is well known that the singlet-triplet energy splitting depends on the amount of Hartree-Fock type exchange in the density functional.[12] BP86 was used in combination with the resolution-of-identity technique to reduce computational costs.[13] The effect of Grimme's empirical dispersion corrections of the Becke-Johnson type was also tested.[14] Solvation was treated implicitly by the conductor-like-screening model (COSMO) as implemented in Turbomole:[15] the solvent is described as a dielectric continuum and the solute is placed in a cavity in this continuum. To match experimental conditions, the dielectric constant of nitromethane is chosen, (ε = 35.9) as well as a dielectric constant of DMSO (ε = 46.7) for sake of comparison. If not noted otherwise, structures are fully optimized.

The molecular electrostatic potential (MEP) was calculated to evaluate the relative polarity and thus the reactivity of the molecule under study, here CcC. It is plotted on a density isovalue of 0.02 a.u., whereas red areas indicate a negative MEP and blue areas a positive MEP.[16]

Proton affinities were calculated as the negative enthalpy $-\Delta H$ of the following reaction in gas phase:

$$CcC(g) + H^+ \leftrightarrow CcCH^+ \quad (1)$$

pK_a values in solution were obtained with an implicit solvent description from the Gibbs free energy of the solvation process $\Delta G^{\mathrm{Rxn}}{}_{(\mathrm{solv})}$ according to

$$\mathrm{p}K_a = \frac{\Delta G^{\mathrm{Rxn}}{}_{(\mathrm{solv})}}{\mathrm{RTln(10)}} \quad (2)$$

with R being the universal gas constant and T denoting the temperature. $\Delta G^{\mathrm{Rxn}}{}_{(\mathrm{solv})}$ cannot be calculated directly but has to be obtained via the thermodynamic cycle depicted in Scheme 1 by calculation of the gas phase reaction free energy $\Delta G^{\mathrm{Rxn}}{}_{(\mathrm{g})}$ and the free energies of solvation for each species, $\Delta G_{(\mathrm{solv})}$ (HA), $\Delta G_{(\mathrm{solv})}$ (H^+), and $\Delta G_{(\mathrm{solv})}$ (A^-). Here, $\Delta G_{(\mathrm{solv})}$ (HA) is $\Delta G_{(\mathrm{solv})}$ (CcCH) and $\Delta G_{(\mathrm{solv})}$ (A^-) corresponds to $\Delta G_{(\mathrm{solv})}$ (CcC).

The gas phase free energy of H^+, $G_{(\mathrm{g})}$ (H^+) = -6.29 kcal/mol, was obtained from the Sackur-Tetrode equation and translational energy at 298 K[17], while for the free energy of solvation, $\Delta G_{(\mathrm{solv})}$ (H^+), the recommended literature value of -259.80 kcal/mol was used.[18]

Tolman Electronic Parameters (TEP) are a simple means to experimentally characterize NHCs and related species in terms of their electronic properties as ligands in organometallic complexes. Quantum chemical TEP calculations of these complexes typically depend, amongst other, on the employed exchange-correlation functional and transferability of the results may not be straight forward. Fortunately, a parameterization scheme has been developed by Mathew and Sureh, which allows for TEP calculations of the ligand alone and which only requires the molecular electrostatic potential at the carbene (V_C).[19] For sake of comparison, calculation of TEPs in this study were performed with Gausian09[20] using the density functionals B3LYP,[21] BP86,[7,8] and M05[22] in combination with the Pople type basis set 6-311+G(d,p) basis set as implemented in Gaussian09.

Structures and MEPs were visualized with PyMol.[23]

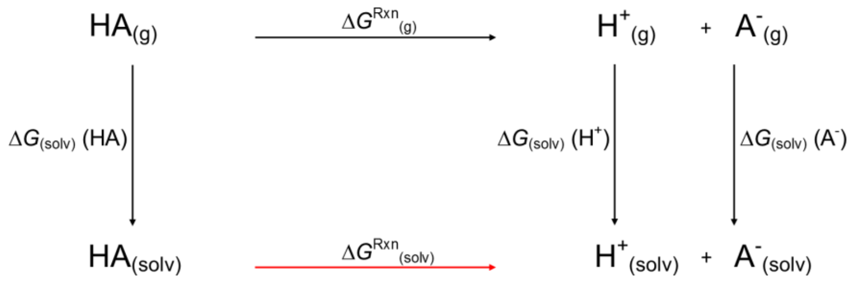

Scheme 1. Thermodynamic cycle used for the calculation of pKa values. While $\Delta G^{\mathrm{Rxn}}{}_{(\mathrm{solv})}$ (depicted by the red arrow) is the property of interest, it has to be calculated via the gas phase reaction free energy and the solvation free energies of each species.

Results Electronic Structure Investigation

Singlet-Triplet Splitting

Singlet-triplet relative energy splittings of fully optimized NHC and CcC were calculated. While we found that CcC shows a significantly smaller singlet-triplet gap as NHC as may be seen from Table S19, individual values depend on the basis set, and more importantly, on density functional employed. The singlet-triplet splitting decreases if a density functional with Hartree-Fock type exchange as the PBE0 density functional with 25% HF exchange is used. Empirical dispersion corrections (denoted as BJ) exert only a minor influence on the energy splittings implying structural effects to be small.

Table S19. Singlet-triplet splitting in kcal/mol of the fully optimized NHC and CcC structures.

**NHC**	$\Delta E_{singlet\text{-}triplet}$/def2-SV(P)	$\Delta E_{singlet\text{-}triplet}$/def2-TZVPP
BP86	84.00	83.03
BP86/BJ	84.03	83.05
PBE0	81.83	81.69
PBE0/BJ	81.84	81.70
**CcC**		
BP86	22.67	25.96
BP86/BJ	23.60	26.89
PBE0	17.21	20.73
PBE0/BJ	18.16	21.32

Structural Parameters

Structural parameters of the ligand CcC and the complex (CcC)Au(CN)$_3$ (both depicted in Figure S27) are listed in Table S20 and S21. These include Co-C and C-C bond lengths, selected bond angles and dihedrals. In the case of CcC, the angle between the two Cp-planes (denoted as $\Theta_{Cp\text{-}Cp}$) as well as the distance of the Co-Cp axis to the center of mass of the upper Cp ($d_{out\text{-}of\text{-}center}$) were also evaluated.

Structural parameters agree well with experimentally determined values for (CcC)Au(CN)$_3$ and small differences may be attributed to packing effects. Coordination of CcC to Au(CN)$_3$ has only a small impact on its structural parameters and Co-C and C-C bond lengths are very similar (compare Table S20 and Table S21).

Concerning CcC, $\Theta_{Cp\text{-}Cp}$ and the distance of the Co-Cp axis to the center of mass of the upper Cp ($d_{out\text{-}of\text{-}center}$) are very small, the two Cp rings are almost perfectly stacked on top of each other, even though the two Cp planes are slightly rotated in (CcC)Au(CN)$_3$.

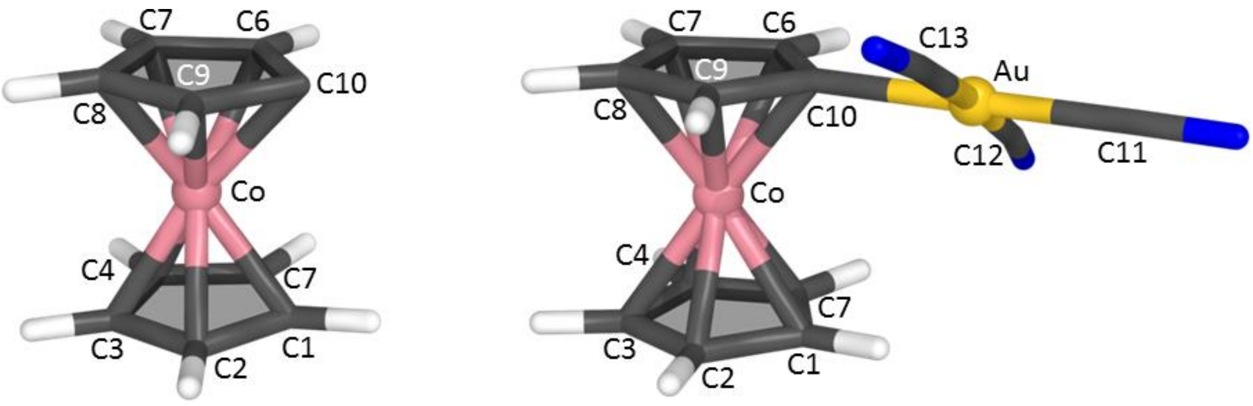

Figure S27. PBE0/BJ/def2-TZVPP optimized structure of CcC and (CcC)Au(CN)$_3$.

Table S20. Structural parameters of CcC for various density functionals plus empirical dispersion corrections. $\Theta_{Cp\text{-}Cp}$ denotes the angle between the two Cp-planes, and $d_{out\text{-}of\text{-}center}$ denotes the distance of the Co-Cp axis to the center of mass of the upper Cp. Angles are given in °, bond lengths and distances in Å.

**CcC**	**BP86/ def2-TZVPP**	**BP86/BJ/ def2-TZVPP**	**PBE0/ def2-TZVPP**	**PBE0/BJ/ def2-TZVPP**
Co-C10	2.114	2.108	2.099	2.096
Co-C6	2.037	2.030	2.014	2.011
Co-C7	2.018	2.012	2.004	2.001
Co-C8	2.018	2.012	2.004	2.001
Co-C9	2.037	2.030	2.014	2.011
C10-C9	1.454	1.454	1.442	1.442
C10-C6	1.454	1.454	1.442	1.442
C9-C8	1.447	1.447	1.431	1.431
C6-C7	1.447	1.447	1.431	1.431
C7-C8	1.432	1.432	1.419	1.419
Co-C5	2.059	2.050	2.042	2.037
Co-C1	2.055	2.046	2.041	2.037
Co-C4	2.065	2.057	2.049	2.036
Co-C3	2.065	2.056	2.050	2.046
Co-C2	2.055	2.046	2.040	2.046
$\Theta_{Cp\text{-}Cp}$	2.731	2.784	2.735	2.784
$d_{out\text{-}of\text{-}center}$	0.157	0.159	0.156	0.160

Table S21. Structural parameters of the optimized (CcC)Au$(CN)_3$ obtained for various density functionals and empirical dispersion corrections denoted as BJ. For sake of comparison, experimental X-ray crystal structure values are also listed.

	**BP86/ def2-TZVPP**	**BP86/BJ/ def2-TZVPP**	**PBE0/ def2-TZVPP**	**PBE0/BJ/ def2-TZVPP**	**X-ray**
Au-C10	2.075	2.065	2.053	2.048	2.038(3)
Au-C11	2.021	2.018	2.005	2.004	2.080(4)
Au-C12	2.012	2.008	1.997	1.995	2.003(4)
Au-C13	2.005	2.000	1.989	1.987	2.013(4)
Co-C10	2.081	2.058	2.059	2.049	2.048(3)
Co-C6	2.043	2.034	2.026	2.021	2.028(3)
Co-C7	2.041	2.037	2.026	2.023	2.029(4)
Co-C8	2.045	2.040	2.029	2.026	2.026(3)
Co-C9	2.054	2.043	2.037	2.032	2.029(3)
C10-C9	1.441	1.441	1.428	1.427	1.425(5)
C10-C6	1.442	1.441	1.429	1.428	1.421(5)
C9-C8	1.432	1.432	1.419	1.419	1.416(5)
C6-C7	1.433	1.432	1.420	1.420	1.415(5)
C7-C8	1.433	1.433	1.420	1.420	1.419(6)
C11-Au-C13	91.29	91.60	91.35	91.51	90.66(16)
C11-Au-C12	91.40	91.54	91.43	91.55	90.99(15)
Plane(C11-C6)(Au$(CN)_3$)	63.24	65.91	63.73	65.03	34.0(1)

Tolman Electronic Parameter

Tolman Electronic Parameter were calculated according to a parametrization by Mathew and Suresh on the PBE0/BJ/def2-TZVPP optimized structures. As may be seen from calculated NHC TEP values (Table S22), they are little functional dependent and within an error range of around 2 cm^{-1} to the experimental value of 2051.2 cm^{-1}. While for CcC no experimental data is available, all calculated TEP are around 2038 cm^{-1} and significantly smaller than those of NHC. To separate structural from electronic effects, a TEP calculation with B3LYP/6-311++G(d,p) was performed on the BP86/def2-TZVPP optimized but a very similar value was obtained and electronic effects account for less than 1 cm^{-1}.

Table S22. Tolman electronic parameter (TEP) for NHC and for CcC. The electrostatic potential V_C is calculated at the carbene atom.

**NHC**	V_c [kcal/mol]	TEP [cm^{-1}]
B3LYP/6-311++G(d,p)	-9279.31	2050.32
BP86/6-311++G(d,p)	-9285.86	2049.22
M05/6-311++G(d,p)	-9283.68	2049.74
Exp.		2051.2
**CcC**	V_c [kcal/mol]	TEP [cm^{-1}]
B3LYP/6-311++G(d,p)	-9309.64	2037.17
BP86/6-311++G(d,p)	-9310.95	2038.01
M05/6-311++G(d,p)	-9311.01	2037.58
B3LYP/6-311++G(d,p)*	-9307.02	2038.31
Exp.		*N/A*

*On BP86/def2-TZVP optimized structure

Proton Affinities and p*K*

Proton affinities and pK_a values are a further characteristic of NHC and related ligands and are also investigated by DFT (Table S23). Proton affinities were calculated according to Eq. (1), whereas pK_a values according to the thermodynamic cycle depicted in Scheme 1. The solvent was described implicitly by a continuum solvation model (see Computational Methodology for details). While for NHC an experimental p*K*a of approx. 24 was found [19], no experimental value is available for CcC.

Although individual pK_a values depend on the density functional employed and – in the case of NHC – differ somewhat from the experimental values, CcC shows significantly higher values between 38 and 40 than NHC. Thus, it is a much stronger base in comparison.

Table S23. Proton affinities in the gas phase as well as pK_a values in DMSO and nitromethane for NHC and CcC.

**NHC**	Proton Affinity [kcal/mol]	pK_a in DMSO	pK_a in Nitromethane
BP86/def2-TZVPP	261.73	26.4	26.1
BP86/BJ/def2-TZVPP	262.35	26.9	26.5
PBE0/def2-TZVPP	263.50	27.8	27.5
PBE0/BJ/def2-TZVPP	263.86	28.1	27.8
Exp.[24]		24*	
**CcC**			
BP86/def2-TZVPP	287.92	38.7	38.5
BP86/BJ/def2-TZVPP	288.43	39.1	39.9
PBE0/def2-TZVPP	291.42	40.6	40.4
PBE0/BJ/def2-TZVPP	291.72	40.5	40.3

References

(1) Vanicek, S.; Kopacka, H.; Wurst, K.; Müller, T.; Hassenrück, C.; Winter, R. F.; Bidstein, B., *Organometallics* **2016**, *35*, 2101–2109.
(2) Seliman, A. A. A., Altaf, M.; Kawde, A.-N.; Wazeer, M. I. M., Isab, A. A., *J. Coord. Chem.* **2014**, *67(21)*, 3431–3443.
(3) Huang, L.; Rominger, F.; Rudolph, M.; Hashmi, A. S. K., *Chem. Commun.* **2016**, *52*, 6435–6438.
(4) Thompson, D. P.; Boudjouk, P., *J. Org. Chem.* **1988**, *53*, 2109–2112.
(5) Krejčik, M.; Daněk, M.; Hartl, F., *J. Electroanal. Chem.* **1991**, *317*, 179–187.
(6) TURBOMOLE V6.6 2014, a development of University of Karlsruhe and Forschungszentrum Karlsruhe GmbH, 1989-2007, TURBOMOLE GmbH, since 2007; available from http://www.turbomole.com.
(7) Becke, A. D., Phys Rev A 1988, 38(6): 3098–3100.
(8) Perdew, J. P., *Phys. Rev. B*, **1986**, 33 (12), 8822–8824.
(9) Perdew J. P.; Burke K.; Ernzerhof, M., *Phys. Rev. Lett.* **1996**, 77 (18), 3865–3868.
(10) Weigend, F.; Ahlrichs, R., *Phys. Chem. Chem. Phys.* **2005**, 7, 3297–3305.
(11) Andrae, D.; Haeussermann, U.; Dolg, M.; Stoll, H.; Preuss, H., *Theor. Chim. Acta* **1990**, 77, 123–141.
(12) Eichkorn, K.; Treutler, O.; Öhm, H.; Häser, M.; Ahlrichs, R., *Chem. Phys. Lett.* **1995**, 240(4), 283–290.
(13) (a) Podewitz, M.; Weymuth, T.; Reiher, M., in Comba, P., Eds., Modeling Molecular Properties, p. 139–163. 2011 (b) Reiher, M.; Salomon, O.; Hess, B. A., *Theor. Chem. Acc.* **2001**, 107, 48–55.
(14) (a) Grimme, S.; Antony, J.; Ehrlich, S.; Krieg, H., *J. Chem. Phys.* **2010**, 132, 154104. (b) Grimme, S.; Ehrlich, S.; Goerigk, L., *J. Comp. Chem.* **2011**, 32, 1456–1465.
(15) Klamt, A.; Schüürmann, G., *J. Chem. Soc. Perkin Trans. 2*, **1993**, (5), 799–805.
(16) (a) Leboeuf, M.; Köster, A. M.; Jug, K.; Salahub, D. R., **1999**, *J. Chem. Phys.* 111, 4893–4905. (b) Molecular Electrostatic Potentials: Concepts and Applications, Murray, J. S.; Sen, K., Elsevier, Amsterdam, 1996, ISBN: 0-444-82353-0.
(17) See e.g., Ho, J.; Coote, M. L., *Wiley Interdiscip. Rev. Comput. Mol. Sci.* **2011**, 1, 649–660, and references therein.
(18) Single-Ion Solvation. Experimental and Theoretical Approaches to Elusive Thermodynamic Quantities. Hünenberger, P.; Reif, M., Royal Society of Chemistry - Theoretical and Computational Chemistry Series London, UK, ISBN: 978-1-84755-187-0 (2011).
(19) Mathew J., Suresh, C. H., *Inorg. Chem.* **2010**, 49, 4665–4669.
(20) Gaussian 09, Revision D.01, M. J. Frisch, G. W. Trucks, H. B. Schlegel, G. E. Scuseria, M. A. Robb, J. R. Cheeseman, G. Scalmani, V. Barone, G. A. Petersson, H. Nakatsuji, X. Li, M. Caricato, A. Marenich, J. Bloino, B. G. Janesko, R. Gomperts, B. Mennucci, H. P. Hratchian, J. V. Ortiz, A. F. Izmaylov, J. L. Sonnenberg, D. Williams-Young, F. Ding, F. Lipparini, F. Egidi, J. Goings, B. Peng, A. Petrone, T. Henderson, D. Ranasinghe, V. G. Zakrzewski, J. Gao, N. Rega, G. Zheng, W. Liang, M. Hada, M. Ehara, K. Toyota, R. Fukuda, J. Hasegawa, M. Ishida, T. Nakajima, Y. Honda, O. Kitao, H. Nakai, T. Vreven, K. Throssell, J. A. Montgomery, Jr., J. E. Peralta, F. Ogliaro, M. Bearpark, J. J. Heyd, E. Brothers, K. N. Kudin, V. N. Staroverov, T. Keith, R. Kobayashi, J. Normand, K. Raghavachari, A. Rendell, J. C. Burant, S. S. Iyengar, J. Tomasi, M. Cossi, J. M. Millam, M. Klene, C. Adamo, R. Cammi, J. W. Ochterski, R. L. Martin, K. Morokuma, O. Farkas, J. B. Foresman, and D. J. Fox, Gaussian, Inc., Wallingford CT, 2009.
(21) Becke, A. D., *J. Chem. Phys.* **1993**, 98 (2), 1372–1377.
(22) Zhao, Y.; Schultz, N. E.; Truhlar, D. G., *J. Chem. Phys.* **2005**, 123, 161103.
(23) The PyMOL Molecular Graphics System, Version 1.8 Schrödinger, LLC.
(24) Alder, R. W.; Allen, P. R.; Williams, S. J., *J. Chem. Soc., Chem. Commun.* **1995**, (12) 1267–1268.
